# Plasma membrane damage limits cytoplasmic delivery by conventional cell penetrating peptides

**DOI:** 10.1371/journal.pone.0305848

**Published:** 2024-09-03

**Authors:** Stéphanie G. I. Polderdijk, Jazeel F. Limzerwala, Christoph Spiess

**Affiliations:** Department of Antibody Engineering, Genentech Inc., South San Francisco, CA, United States of America; University of Helsinki, FINLAND

## Abstract

Intracellular delivery of large molecule cargo via cell penetrating peptides (CPPs) is an inefficient process and despite intense efforts in past decades, improvements in efficiency have been marginal. Utilizing a standardized and comparative analysis of the delivery efficiency of previously described cationic, anionic, and amphiphilic CPPs, we demonstrate that the delivery ceiling is accompanied by irreparable plasma membrane damage that is part of the uptake mechanism. As a consequence, intracellular delivery correlates with cell toxicity and is more efficient for smaller peptides than for large molecule cargo. The delivery of pharmaceutically relevant cargo quantities with acceptable toxicity thus seems hard to achieve with the CPPs tested in our study. Our results suggest that any engineered intracellular delivery system based on conventional cationic or amphiphilic CPPs, or the design principles underlying them, needs to accept low delivery yields due to toxicity limiting efficient cytoplasmic uptake. Novel peptide designs based on detailed study of uptake mechanisms are required to overcome these limitations.

## Introduction

Cell penetrating peptides (CPPs) have been extensively studied over the past three decades for the delivery of diverse macromolecules into the cytoplasm of cells, including peptides, proteins, nucleic acids, and small molecules [1, 2]. However, despite these efforts translation of CPPs from an *in vitro* setting to preclinical *in vivo* studies and clinical applications has been very limited (for recent reviews, see [3, 4]). Major obstacles include the requirement for a high concentration of CPP, non-specific membrane targeting, low uptake efficiency (< 10% in vitro), toxicity, and reduced efficacy when fused to a cargo.

CPPs were initially discovered as part of viral proteins [5, 6] or transcription factors [7]. A wide range of peptides has since been described and designed for use as cell-targeting molecules and efforts to improve their delivery or attenuate their toxicity have been reported [8]. Some examples include reduced toxicity derivatives of naturally occurring toxin peptides [9–11], fusions of naturally occurring CPPs (Transportan [12]) or the development of fully synthetic sequences (TP3 [13, 14]).

A major issue with identifying and comparing peptides for different applications remains the lack of standardized assay techniques for identifying true cytoplasmic delivery. Early comparative studies often employed total cell lysate or flow cytometry, which quantify total cell uptake but disregard the cellular localization of the CPP. This can lead to overestimation of delivery efficiency as plasma membrane association and uptake of positively charged peptides through pinocytosis and their entrapment in endosomes can be misinterpreted as successful cytoplasmic delivery. Additionally, the widespread use of fixed cell microscopy to determine cytoplasmic delivery has been shown to introduce substantial artifacts in pinpointing the cellular localization, overestimating the amount of material delivered to the cytoplasm [15]. Deprey et al. provide a recent comprehensive overview of historical assay methods and their drawbacks [16].

Newer approaches, employing split protein complementation assays, BirA-based tagging of delivered protein [17–19], live-cell confocal [20] or FCS microscopy [21] have suggested that only a small percentage of total peptide taken up by cells is transferred to the cytoplasm, indicating that delivery has been overestimated historically.

In this study, we addressed several of these limitations by studying a defined set of previously reported CPPs. Their internalization efficiency was measured using a sensitive split-Nanoluciferase assay [22]. For CPP-HiBiT fusions, we compared relative delivery with or without CPP to assess improvement mediated by the CPP fusion. Total delivery was estimated in a subset of experiments by comparing signal in live cells with total signal obtained after cell lysis to yield relative delivery, revealing that delivery efficiency is low and generally in the single digit percentage range. We additionally evaluated peptide toxicity at different concentrations and timepoints to obtain a relationship between concentration, incubation time and delivery efficiency, showing that initial high delivery is often accompanied by substantial toxicity, caused by irreversible plasma membrane damage. The CPP efficacy is reduced when the cargo size is increased as evaluated by delivery of Fab fragments by CPPs. Fusion of CPPs to fragment antigen-binding (Fab) fragments similarly only produced a moderate increase in cargo uptake, indicating that delivery efficiency seen for smaller cargoes, such as dyes and small peptides, does not translate to larger, more complex, cargoes.

## Materials and methods

### Reagents

Peptides were synthesized using L-amino acids by Elim Biopharm (≥ 90% purity for experiments showing HiBiT fusions to CPP, ≥ 95% for isolated CPPs in the *trans* deliver experiments) with N-terminal acetylation and C-terminal amidation.

### Cell culture

For regular cell maintenance, HeLa cells were grown in Complete media (Dulbecco’s Modified Eagle Medium (DMEM) powder (Corning, #50-013-PC), 3.7 g/L sodium bicarbonate, 0.11 g/L sodium pyruvate with 10% fetal bovine serum (FBS) and 2 mM L-Gln added) at 37˚C and 5% CO_2_.

HeLa cells stably expressing LgBiT were generated using the Piggybac transposon system. Briefly, cells were co-transfected with a vector encoding Piggybac transposase, as well as a vector containing the LgBiT coding sequence (see supporting information for the protein sequence), controlled by a tet-inducible TRE3GS promoter, as well as an IRES preceding an eGFP coding sequence for easy identification of positive clones. Cells were selected in selection media (= Complete media + 2 μg/ml puromycin dihydrochloride (Thermo Fisher, #A1113803)). After selection, cells were induced in induction media (= Selection media + 0.25 μg/ml doxycycline (Takara Bio)) and bulk sorting was performed to isolate GFP positive cells. Bulk pools were frozen and used in all luciferase complementation experiments.

### Protein expression and purification

The coding sequence for LgBiT [22] was cloned into in-house expression vector pST239 with a *phoA* promoter, and N-terminal double HQ-tag for purification [23]. LgBiT was expressed in *E*. *coli* 58F3 (derived from W3110 with genotype Δ*fhuA (ΔtonA) Δlon galE rpoHts(htpRts) ΔclpP lacIq ΔompT Δ(nmpc-fepE) ΔslyD*) essentially as described [24]. After 24 h at 30˚C, cultures were harvested by centrifugation and the pellet frozen at -20˚C until processing.

Cell pellets were resuspended in 20 mM Tris pH 7.5, 500 mM NaCl with added Complete Protease inhibitor (Roche, #5892970001) and Lysonase Bioprocessing reagent (Sigma, Millipore, #71230). Cells were lysed using a microfluidizer (Microfluidics Corp., Newton, MA) and the lysate cleared by centrifugation. After filtering through an 0.22 μm filter, the lysate was applied to a HisTrap Excel column (Cytiva, 17371206), equilibrated in 20 mM Tris pH 7.5, 500 mM NaCl, washed with equilibration buffer with 5 mM imidazole and the protein eluted with a 5 mM-250 mM imidazole gradient. Fractions containing LgBiT were pooled and dialyzed into phosphate buffered saline (PBS) pH 7.4. Protein concentration was determined by absorbance at 280 nm, using an absorbance of 1.0 for a 0.1% solution.

### Expression and purification of Fab and Fab-CPP fusions

Fab fragments targeting either herpes simplex virus (HSV-1) gD-protein (anti-gD [25]) or the human transferrin receptor (TfR) (anti-TfR^1^ [26]) with a heavy chain (HC) C-terminal HiBiT tag, separated from the Fab by a single GSGG spacer, were expressed from Expi293 cells at 30 ml scale. Single-step purification was done using CaptureSelect CH1-XL resin (Thermo Fisher). For CPP fusions, CPPs were added at either the antibody light chain (LC) C-terminus, the LC N-terminus, or the heavy chain (HC) N-terminus, in all cases with a single GGA spacer between the CPP and LC sequences.

### LgBiT complementation assay with purified proteins

Peptides and purified LgBiT were diluted into assay buffer (PBS + 0.05% v/v Tween20) to 2x final assay concentration and mixed 1:1 in a white, opaque, flat bottom, 96-well plate (Corning, 3610). Samples were incubated at 37˚C for 30 min to allow complementation to occur. To read out the assay, Nano-Glo Live Cell substrate (Promega, #N2011) was added as per manufacturer’s instructions and luminescence read using an EnVision 2104–0020 Multilabel Plate Reader (Perkin Elmer). Assays were run three times, with different concentrations of HiBiT fusion. Each set of concentrations was run twice. Final assay concentration for LgBiT was 5 nM. To determine EC_50_, luminescence was normalized to the HiBiT control and fits analyzed in Graphpad Prism 9.4, using a nonlinear regression fit ([Agonist] vs. response, three parameters).

### Internalization assays by luciferase complementation

HeLa cells, stably transfected with a doxycycline inducible LgBiT reporter were plated at 10,000 cells/well in a 96-well, opaque flat bottom plate and left to attach overnight in selection media. The next day, reporter expression was induced with 0.25 μg/ml doxycycline. After overnight incubation, cells were washed with assay buffer (Hank’s balanced salt solution (HBSS), CellGro #55–022 + 0.35 g/L sodium bicarbonate + 0.5 mM MgCl_2_ + 1.2 mM CaCl_2_ + 25 mM D-Glucose + 10 mM HEPES pH 7.5 + 2 μg/ml puromycin dihydrochloride (Thermo Fisher, #A1113803) + 0.25 μg/ml doxycycline (Takara Bio)), and peptides and proteins of interest, diluted in assay buffer, were added. After incubation for the time indicated in the figure legends, the media was removed, the cells were washed three times with either assay buffer (for HBSS experiments) or PBS (for experiments in media) and fresh assay buffer or PBS was added before addition of NanoGlo live cell substrate (Promega, #N2011). Luminescence was read using an EnVision 2104–0020 Multilabel Plate Reader (Perkin Elmer). After luminescence readout, cells were washed again and fresh selection media was added. Cells were incubated overnight at 37˚C, 5% CO_2_ and toxicity was assessed by a Cell-Titer Glo (CTG) assay the next day (Promega, #G9241).

### Assessment of LgBiT leakage and re-internalization

HeLa LgBiT reporter cells were prepared and induced as described above. On the second day of the assay, HeLa cells not expressing reporter, were also plated at 10,000 cells/well and left to attach overnight. The next day, HeLa LgBiT reporter cells were incubated with indicated concentrations of peptides for 30 min or 1 h. After incubation, the media was removed, the cells washed three times with PBS, before reading luminescence as above. The conditioned media was transferred to the plate containing HeLa cells and the cells incubated for 30 min or 1 h. The media was removed, cells washed three times with PBS and luminescence read as above. Additionally, the media was transferred to a fresh plate and NanoGlo live cell substrate added to read luminescence in the media.

### Membrane damage measurement by propidium iodide fluorescence

Membrane damage assays were essentially performed as described [27]. Briefly, HeLa cells were plated the day prior to the experiment at 10,000 cells/well in complete media in 96-well black, clear bottom microplates (Corning, #3603) and left to attach overnight. For assays including calcium, the cells were washed in M1 buffer (HBSS, CellGro #55–022 + 0.35 g/L sodium bicarbonate + 0.5 mM MgCl_2_ + 1.2 mM CaCl_2_ + 25 mM D-Glucose + 10 mM HEPES pH 7.5). For assays not including CaCl_2_, cells were washed in M2 buffer (HBSS, CellGro #55–022 + 0.35 g/L sodium bicarbonate + 0.5 mM MgCl_2_ + 25 mM D-Glucose + 10 mM HEPES pH 7.5) + 5 mM EDTA before washing with M2 buffer. The assay was started by adding (in either M1 or M2 buffer as indicated) the respective peptide dilutions as indicated and propidium iodide (Thermo Fisher, #P3566) to a final concentration of 30 μM. Plates were placed in a SpectraMax M2 or SpectraMax M2e plate reader (Molecular Devices) and fluorescence read for 1 h (excitation = 535 nm, emission = 615 nm, gain = High, 6 flashes/read) at 37˚C. Plots show mean fluorescence, with a dimethyl sulfoxide (DMSO) only control subtracted, from three independent experiments, error bars show the standard error of the mean (SEM).

## Results

### Peptide selection and design of a screening platform utilizing HiBiT fusions

CPPs have been described to enter cells by a variety of mechanisms, including pore formation in the plasma membrane, direct translocation by other mechanisms from the extracellular space into the cytoplasm, and escape from the endosome after internalization (for reviews, see [28]). Some peptides, such as multi-arginine containing peptides, have been reported to utilize all of these mechanisms depending on the peptide concentration used. We hypothesized that different cargoes might require different mechanisms to be delivered efficiently, depending on their localization, charge and size. We therefore selected peptides representing different reported mechanisms and both linear, charged as well as amphipathic/helical peptides as shown in Table 1. Additionally, for some peptides, such as m-Lycotoxin and the melittin-derived peptide MelP5, we included engineered variants that have been reported to show reduced toxicity and increased efficacy as well as being engineered to escape the endosome only after acidification.

**Table 1:** CPP sequences evaluated in this study.

Name	Sequence	Reported mechanisms	Charge (pH 7.5, pH 6)	% positive residues (pH 7.5, pH 6)	References
OctoR	Ac-RRRRRRRR-NH2	Endocytosis (macropinocytosis + other pathways), direct translocation	+8, +8	100%	[29]
P16 (Penetratin)	Ac-RQIKIWFQNRRMKWKK-NH2	Macropinocytosis, direct translocation	+7, +7	43.80%	[30]
L17E	Ac-IWLTALKFLGKHAAKHEAKQQLSKL-NH2	Macropinocytosis, membrane ruffling, pH-dependent endosome escape	+4, +6	20%, 28%	[11, 31]
Lyco	Ac-IWLTALKFLGKHAAKHLAKQQLSKL-NH2	Membrane damage	+5, +7	20%, 28%	[11]
TP10 (Transportan)	Ac-AGYLLGKINLKALAALAKKIL-NH2	Direct translocation	+4, +4	19%	[12]
MelP5	Ac-GIGAVLKVLATGLPALISWIKAAQQL-NH2	Pore formation	+2, +2	7.70%	[9]
pHD24	Ac-GIGDVLHELAADLPELQEWIHAAQQL-NH2	pH-dependent pore formation	-5, -3	0%, 7.7%	[32]
TP3	Ac-RRILLQLLRGQF-NH2	Direct translocation	+3, +3	25%, 25%	[13]
NRTN	Ac-GAAEAARVYDLGLRRLRQRRRLRRERVRA-NH2	Cell surface interaction	+9, +9	37.9%, 37.9%	[13, 33]

Blue = cationic; Red = anionic; Grey = amphiphilic. Charge is shown at physiological pH (7.5) and slightly acidic early endosomal pH (6). Charge differences are due to protonation of His at lower pH.

To assess cytoplasmic delivery, we employed a split Nanoluciferase complementation assay. Briefly, engineered Nanoluciferase (NanoBiT) from deep sea shrimp (*Oplophorus gracilirostris*) was split into two fragments, a larger LgBiT fragment (18 kDa) and the HiBiT peptide (1.3 kDa), as described [22]. These two fragments can self-associate with pM affinity when present in the same compartment and complement each other to form functional Nanoluciferase. A reporter HeLa cell line expressing cytoplasmic and nuclear LgBiT was incubated with cargo fused to HiBiT or HiBiT alone. Successful entry of HiBiT-fused cargo into the cytoplasm (or nucleus) should lead to complementation and luciferase activity, whereas entry of the cargo into the endosome should not result in signal.

In this work we distinguish two different modes of delivery mediated by CPPs: 1) delivery in *trans*, where the CPP is not covalently connected to the cargo and is instead co-incubated and 2) delivery in *cis*, where the CPP is covalently attached to its cargo. To assess the ability of CPPs to deliver a small cargo in *trans* and in *cis*, we synthesized isolated CPPs and also N- and C-terminal peptide fusions of these CPPs with the HiBiT peptide (CPP-HiBiT and HiBiT-CPP, respectively; see Table 1 for CPP sequences). The ability of the different HiBiT constructs to functionally complement LgBiT was first verified with purified components only (Table 2, S1 Fig). For CPP-HiBiT fusions, EC_50_ were similar to HiBiT alone, with a general 1.5–2 fold reduction in the maximum luminescence obtained. For HiBiT-CPP fusions there was an impact on the EC_50_ of around 2–3 fold, indicating that complementation is slightly impacted if HiBiT is fused to the N-terminus of the cargo. Both orientations showed a reduction in the maximum luminescence obtained, indicating that the CPP fusion potentially impacted activity of the complemented enzyme. Since the impairment was largely similar between the different CPPs, we did not correct for this complementation deficiency in subsequent experiments.

**Table 2:** Purified LgBiT complementation by fusion peptides.

	CPP-HiBiT				HiBiT-CPP			
*CPP*	EC_50_	95% CI	Max RF	95% CI	EC_50_	95% CI	Max RF	95% CI
OctoR	14.65	9.24–23.96	0.58	0.53–0.64	20.62	13.70–31.86	0.64	0.59–0.70
P16	8.166	5.70–11.70	0.5691	0.54–0.60	18.27	13.39–25.24	0.5873	0.55–0.63
L17E	9.68	7.08–13.24	0.5957	0.56–0.63	39.51	28.34–55.52	0.7417	0.68–0.81
Lyco	11.21	8.01–15.77	0.5534	0.52–0.59	29.19	21.04–41.01	0.5308	0.49–0.57
TP10	17.91	12.04–27.08	0.5043	0.46–0.55	21.05	15.23–29.45	0.5532	0.51–0.60
MelP5	14.81	10.46–21.20	0.6373	0.59–0.68	40.84	14.89–129.7	0.5525	0.44–0.76
pHD24	2.727	1.76–4.14	0.7889	0.75–0.83	5.925	3.87–8.97	0.8072	0.75–0.86
TP3	12.85	8.23–20.31	0.6159	0.57–0.67	21.39	15.92–29.03	0.7598	0.71–0.81
NRTN	8.085	5.65–11.56	0.6557	0.62–0.70	25.63	15.76–43.57	0.6736	0.61–0.75
	EC_50_		95% CI		Max RF		95% CI	
HiBiT only	9.12		7.21–11.53		1.03		0.98–1.07	

EC_50_ and maximum relative luminescence values (Max RF) for *in vitro* complementation of purified CPP fusions to HiBiT. Relative luminescence is calculated with respect to the maximum value for the HiBiT peptide only (no fusion). Errors shown are the 95% confidence intervals.

### Screening of CPP fusions for internalization over time

**Fig 1:**
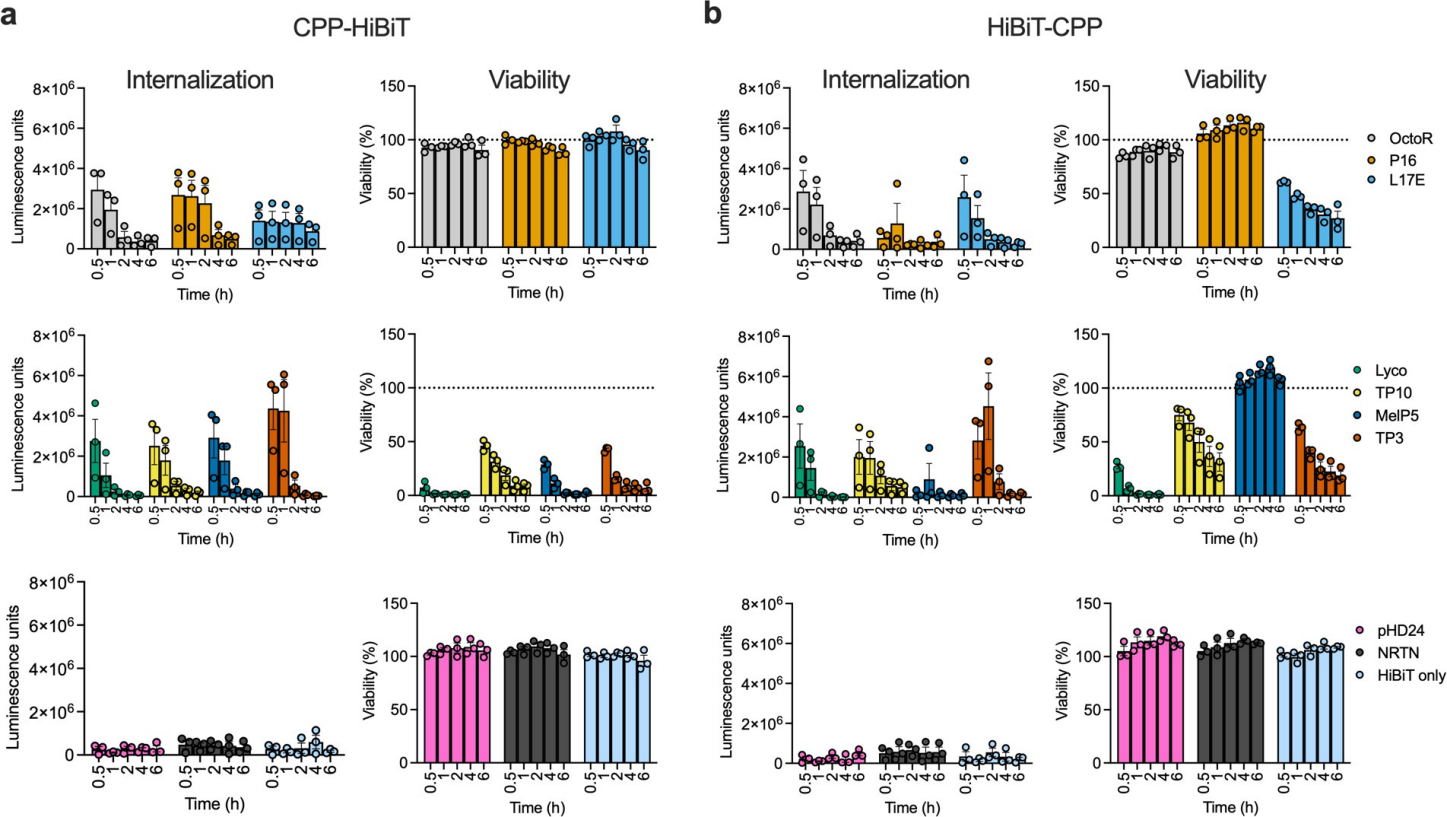
Initial screen of HiBiT fusion peptide internalization in HeLa cells. For each CPP fusion peptide, internalization, as measured by the nanoluciferase complementation assay is shown on the left and viability, as measured by the Cell-TiterGlo assay on the right. Internalization is calculated by subtracting the luminescence for the average of “cells only” samples from all values. Viability is calculated relative to the average of the “cells only” wells. For all graphs, *n* = 3, bars show the mean, error bars show the SEM, individual replicates are shown as symbols. The legends are shown on the right of the Figure and are consistent for all graphs. Top row shows group 1 (internalization, low toxicity), middle row shows group 2 (internalization, high toxicity), bottom row shows group 3 peptides (no internalization, low toxicity). (a) CPP-HiBiT fusions, (b) HiBiT-CPP fusions. Peptide concentration for all experiments is 10 μM.

After confirming complementation with purified components in a cell-free system, we performed an initial screen of the fusion peptides by measuring delivery into the cytoplasm of HeLa cells in media supplemented with 10% FBS. We used a fixed peptide concentration and determined internalization at different incubation times. To assess toxicity, the cells were washed, the media replaced, and the cells incubated overnight before measuring viability using a CellTiter-Glo assay (CTG). Results for all peptides are shown in Fig 1.

The CPPs could be grouped into three distinct categories based on their cell internalization and toxicity properties in this assay: 1) CPPs that mediated internalization without apparent toxicity at 10 μM (Fig 1, top row), 2) CPPs that mediated internalization but showed toxicity (Fig 1, middle row) and 3) CPPs that did not appear to mediate internalization and also showed no toxicity (Fig 1, bottom row). Group 1 includes peptides such as OctoR (displays similar properties as TAT [20]), P16 and L17E. Group 2 comprised Lyco, TP10, MelP5 and TP3. HiBiT alone, pHD24 and NRTN fell into the third group.

The orientation of the fusion peptide did not change their behavior, with the exception of MelP5 and L17E. However, for the MelP5-HiBiT fusion, which did not internalize or show toxicity, we observed significant aggregation in solution prior to the experiment, indicating that this difference may be due to a reduced active concentration in the assay. Interestingly, for most CPPs we observed a strong time dependence of the internalization with almost no peptides showing any increase in luminescence after 2 hrs. This can be explained for the CPPs in group 2 by the profound drop in viability after 2 hrs. For the peptides in group 1, we speculate that it is attributed to rapid intracellular degradation of the internalized peptide, as has been observed previously for Arginine-rich peptides [34]. Based on these results we measured internalization after 30 minutes in all subsequent experiments.

### Extracellular LgBiT is not a source of internalized luciferase signal

**Fig 2:**
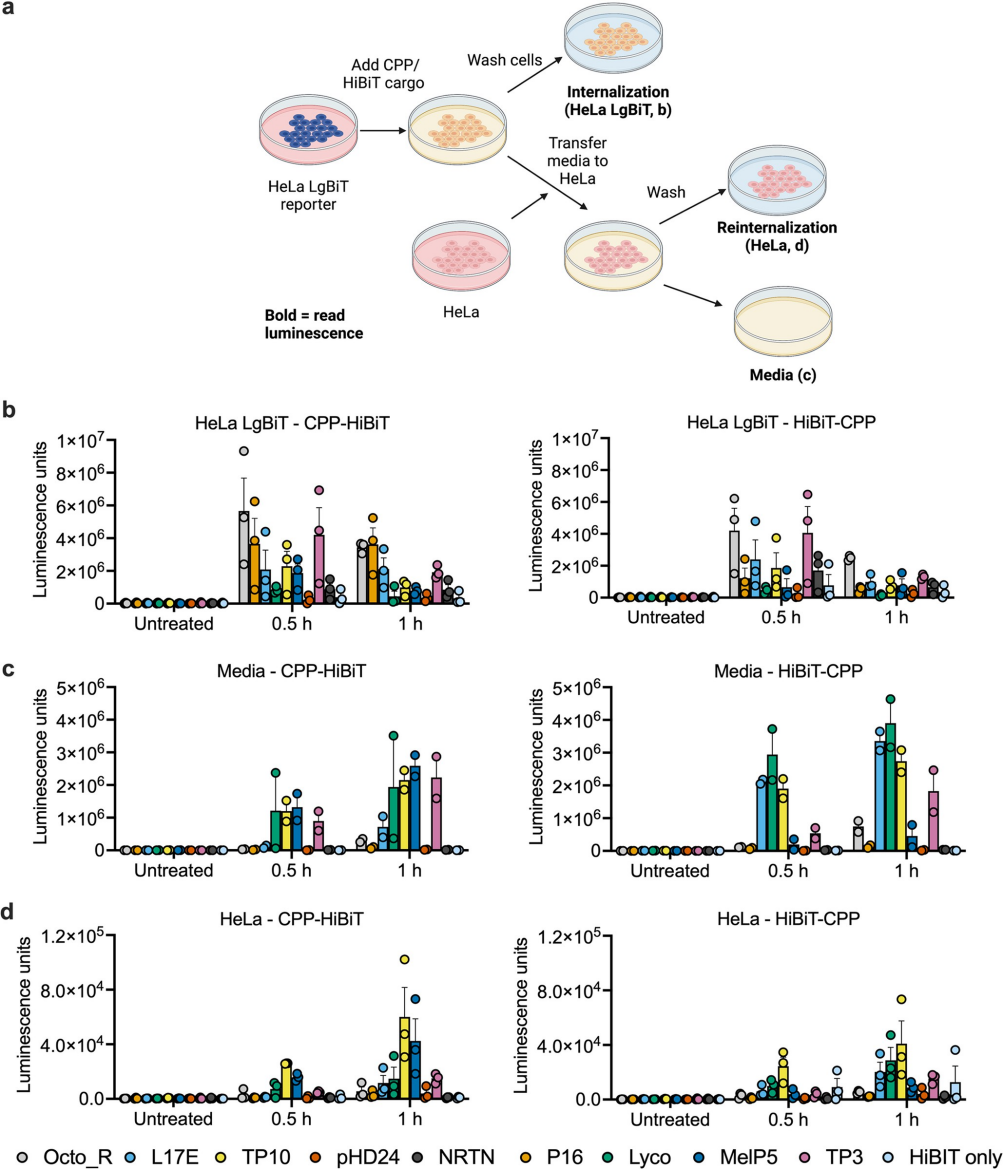
Assessing reinternalization of secreted LgBiT. (a) Overview of the assay. Created with BioRender.com. (b) Internalization of CPP fusions to HiBiT into HeLa LgBiT reporter cells. Peptide concentration in all assays was 10 μM. *n* = 3 (c) Luminescence in the media after the experiment in b. This luminescence represents LgBiT released from reporter cells, complemented by the HiBiT fusions in the media. *n* = 2 (d) Luminescence after incubation of the media from the experiment in C with HeLa cells not expressing the LgBiT reporter, to measure reuptake of complemented LgBiT from the culture media. *n* = 3 for all experiments, bars show the mean, error bars show the SEM. Individual replicates are shown by symbols. CPP-HiBiT fusion peptides are shown on the left, HiBiT-CPP fusions on the right. Note that the scale of the y-axis in (d) is significantly smaller than (b, c).

Previous studies indicate that for most CPPs the majority of uptake occurs by non-specific endocytosis and the CPP is subsequently trapped in endosomes rather than escaping the endosome and reaching the cytoplasm. It has also been reported that part of the CPP mechanism involves adsorption to the cell surface, stimulating uptake [35]. Since we observed significant toxicity for some of our peptides, we investigated if LgBiT that leaks from cells damaged by the CPP may complement the HiBiT peptides in the incubation media. Complemented LgBiT could then potentially be non-specifically endocytosed with the help of the CPP, leading to luciferase signal from the endosome rather than the cytoplasm, artificially increasing the luciferase signal and overestimating true cytoplasmic internalization. To test this possibility, we designed an assay to measure reinternalization (Fig 2A). Internalization was observed for group 1 and group 2 peptides into HeLa LgBiT, as seen in the previous experiment (Fig 2B). The toxic group 2 CPPs additionally increased the luminescence in the cell media (Fig 2C), compared to non-toxic peptides from group 1. This indicates that the cell death was accompanied by membrane damage that allowed for escape of the LgBiT into the extracellular space. However, luminescence from the secondary cells after transfer of the conditioned media remained low for both categories of peptide (Fig 2D, note the differences in the y-axis scale). This confirms that uptake of complemented LgBiT from the media is not a major source of background signal for the peptides tested here.

### Serum-containing media reduces internalization and delivery by CPPs

CPPs are commonly tested in serum-free media, or in buffered solutions without serum. Serum has been reported to reduce toxicity of positively charged peptide surfaces through adsorption of serum proteins onto the positive surface [36]. Additionally, interaction of arginine-rich peptides with serum components has been shown to negatively affect CPP uptake [37]. To evaluate if this also applied to our panel of CPPs, we tested internalization and effect on viability for our CPP panel in serum containing media and serum-free buffer (S2 and S3 Figs). For all peptides tested, except pHD24, we observed a reduction in internalization and toxicity in serum-containing media, indicating that non-specific binding to serum components probably reduced the effective CPP concentration, consistent with results reported by Trofimenko *et al*. [38]. Studies showing internalization of charged CPPs in serum-free media are therefore likely to overestimate the delivery efficiency of the molecules *in vivo*.

### Internalization correlates with toxicity of CPPs

**Fig 3:**
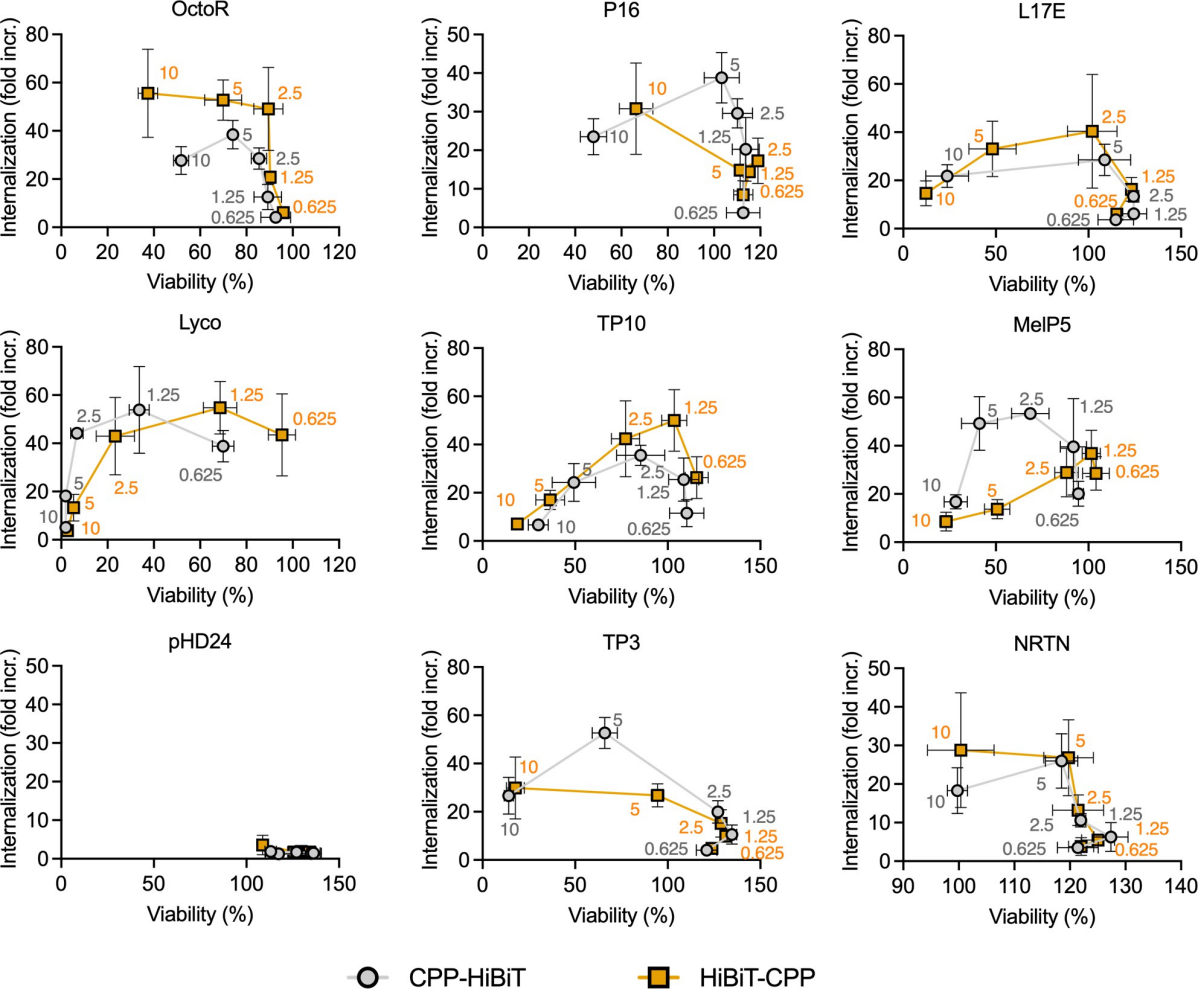
Correlations between internalization and toxicity for CPPs fused to HiBiT in HBSS. Symbols are the mean of three independent experiments, error bars show the SEM. Internalization is calculated as a fold change over the equivalent concentration of HiBiT peptide only. Viability is calculated relative to the average of all “cells only” wells. Each symbol represents a different concentration of peptide used and lines connect the concentrations from high to low. Data points are labeled with the peptide concentration in μM. Data was acquired after 30 minutes incubation of cells with CPP fusions.

To better visualize the relationship between delivery and toxicity of our CPP panel, we plotted the data described in the previous section relative to both internalization and viability. These results are shown in Fig 3. For all peptides, except pHD24, where we observed no internalization at any of the concentrations tested, there appeared to be an optimal concentration that balanced the delivery efficiency with toxicity. This optimal concentration varied depending on the CPP and was generally located within a small window, after which either delivery dropped off, or toxicity increased significantly. Combined with the results from Fig 1, we anticipate that this optimal concentration also shifts with incubation time.

**Fig 4:**
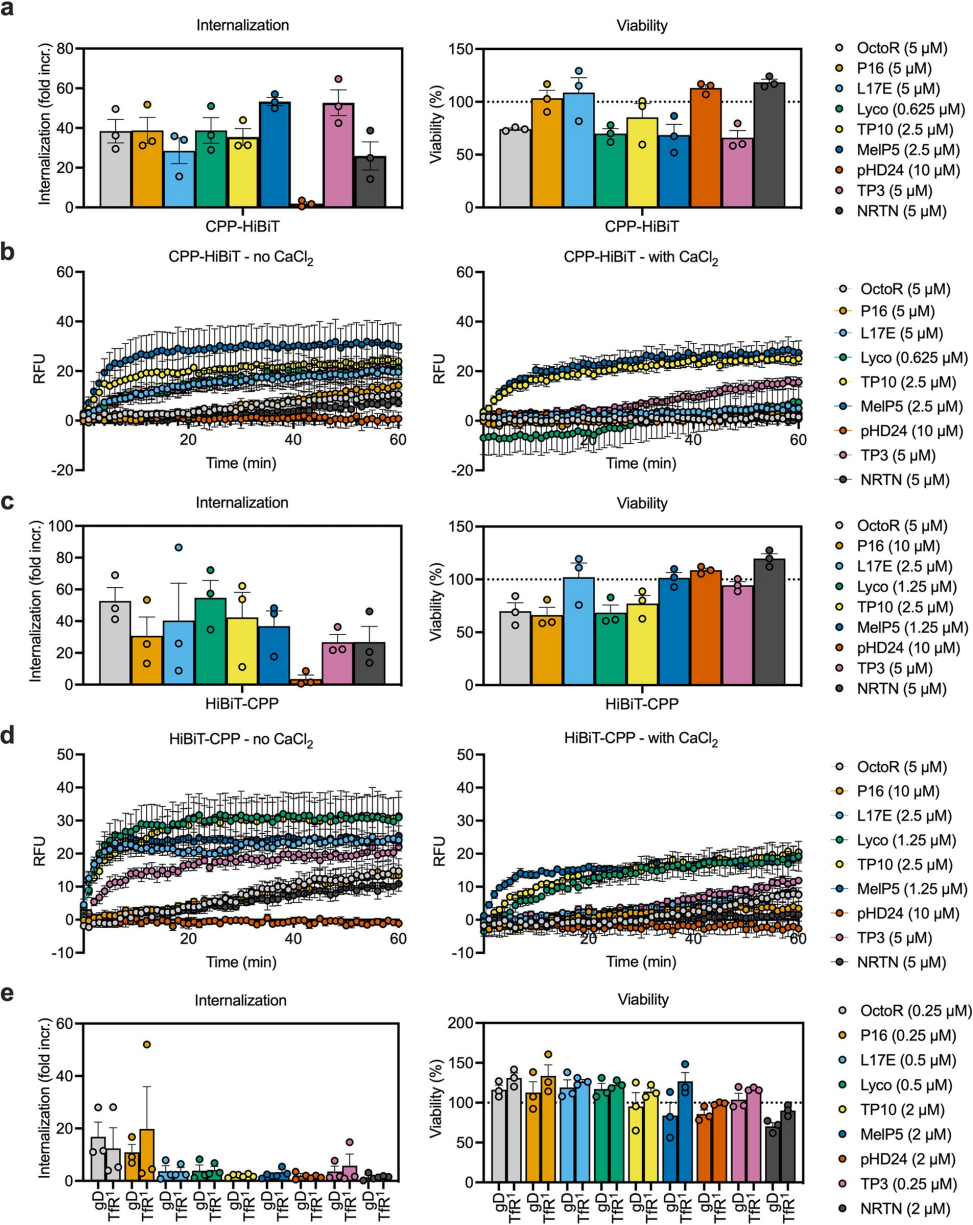
Delivery of cargoes by CPPs in *cis* relies on membrane damage. (a, c) Delivery of different HiBiT to CPP fusions after 30 min incubation. The concentration giving maximum delivery with submaximal toxicity is shown. with CPP concentrations indicated in the legend. (a) shows fusion peptides with the CPP on the N-terminus, (c) shows C-terminal CPP fusions. Internalization is expressed as a fold increase over the equivalent concentration of HiBiT peptide only. Viability is calculated relative to the average of all “cells only” samples. *n = 3*, bars show the mean, individual replicates are shown as symbols, error bars show the SEM. (b, d) Propidium iodide internalization into HeLa cells on incubation with different CPP fusions either in the absence (left) or presence (right) of calcium, to either inhibit or permit plasma membrane repair. Symbols show the mean of at least three replicates, error bars show the SEM. (b) shows N-terminal CPP fusions, (d) shows C-terminal fusions. (e) Internalization of anti-gD or anti-TfR Fab fragments fused to CPPs with the CPP located at the C-terminus of the LC. Internalization is shown relative to Fab without CPP. Viability is calculated relative to the average of all “cells only” samples. *n* = 3, bars show the mean, error bars show the SEM, individual replicates are shown as symbols. Data in a and c is from the same experiment as the experiment shown in Fig 3. The data is reproduced here to allow side-by-side comparison between CPPs. For all concentrations tested per CPP and experiment, see S5–S13 Figs. Data shown here is replicated in those figures for comparison.

To allow a comparison between the CPPs screened, we picked what appeared to be the optimal concentration from the results in Fig 3 and compared the CPP results side-by-side (Fig 4). Additionally, we were interested in the mechanism by which this toxicity occurs. For the full range of concentrations tested for each peptide, see results by peptide in S5 through S13 Figs.

Since these HiBiT fusions to the CPPs were not targeted in any way towards a receptor or other actively internalizing target, we first looked at plasma membrane damage. To evaluate this, we used a propidium iodide (PI) internalization assay, as previously described [27]. Briefly, an increase in PI fluorescence, which can be decreased by the addition of calcium to the culture media, indicates damage to the plasma membrane. Calcium is essential for plasma membrane repair. Comparing PI internalization in the presence (repair) and absence (repair inhibited) of calcium shows the mitigation of membrane damage by plasma membrane repair over time. At the optimal concentration for delivery, all CPPs that showed internalization caused some degree of plasma membrane damage (Fig 4B and 4D). Another interesting observation was that for all CPPs where we observed delivery, this optimal delivery was roughly equal between all CPPs and was not dependent on orientation of the fusion peptide (Fig 4A and 4C). Membrane damage and the concomitant toxicity observed limited delivery mediated by all peptides tested.

Detoxified, engineered variants of naturally occurring toxin peptides, such as L17E (parental peptide: Lyco) and pHD24 (parental peptide: MelP5) either showed no internalization (pHD24), or showed a shifted profile, where both internalization and toxicity were reduced. For L17E we observed similar delivery and toxicity as for Lyco, if the concentration was increased 8-fold. At lower concentrations L17E peptide fusions showed reduced toxicity, but also reduced internalization.

We hypothesized that, while plasma membrane damage might be a major contributor to non-specific delivery, targeted delivery of CPP fusions to the endosome might result in more specific and less toxic endosome escape. We therefore prepared CPP fusions to Fab fragments, targeting either the HSV-1 gD protein (non-binding control) or transferrin receptor (TfR) [26]. We used the non-binding anti-gD Fab to evaluate the effect of increasing the cargo size alone, while the TfR Fab should bind to the transferrin receptor and be actively internalized, increasing targeted delivery of the fusion to the endosome.

We generated CPP fusions either to the N- or C-terminus of the light chain (LC), or to the N-terminus of the heavy chain (HC). The C-terminus of the HC was fused to HiBiT for detection in all Fabs tested. When these were expressed in Expi293 cells, we observed very low yields for HC N-terminal fusions and most N-terminal LC fusions (S14 Fig). C-terminal LC fusions showed good expression and we therefore initially tested internalization by C-terminal LC CPP fusions only. Due to limitations in protein amount and concentration, the highest concentration tested was 2 μM, compared to the 10 μM used for the CPP HiBiT fusion peptides.

Internalization of Fab-CPP fusions was low for all CPPs (Fig 4E). The only CPPs showing a slightly increased internalization were OctoR and P16. None of the fusions affected viability and there was no difference between the targeted and non-targeted Fab, indicating that endosomal delivery and escape likely have a minor contribution compared to the direct delivery mediated by plasma membrane damage. Interestingly, fusion to Fab appeared to reduce toxicity of the CPPs. Both MelP5 and Lyco showed significant toxicity as HiBiT fusions at 2.5 μM (S8G and S10G Figs), whereas the Fab fusions showed no toxicity at the maximum concentration tested (2 μM, S8H and S10H Figs).

The differences in expression between the different fusions of CPP to Fab fragments led us to question if reduced expression might have been due to re-uptake of the secreted Fab fragments into the expression host cells. Especially since the only CPP that had not shown any uptake in our experiments thus far, pHD24, showed little difference in expression between the different CPP Fab orientations. Therefore, for those Fab fusions where we obtained some material, we compared internalization and toxicity of N- and C-terminal CPP Fab fusions (S15 and S16 Figs).

The results for this experiment were CPP-dependent. For P16, L17E, Lyco and TP10 fusions, we saw increased internalization for the N-terminal fusions, consistent with their reduced yields from expression and purification. However, for other CPPs, such as MelP5 and TP3, there was no difference despite the yields for N-terminal fusions being much reduced. For OctoR and NRTN we did not obtain sufficient N-terminal material to perform the experiments.

### Delivery of cargoes by CPPs in trans is mediated by plasma membrane damage

**Fig 5:**
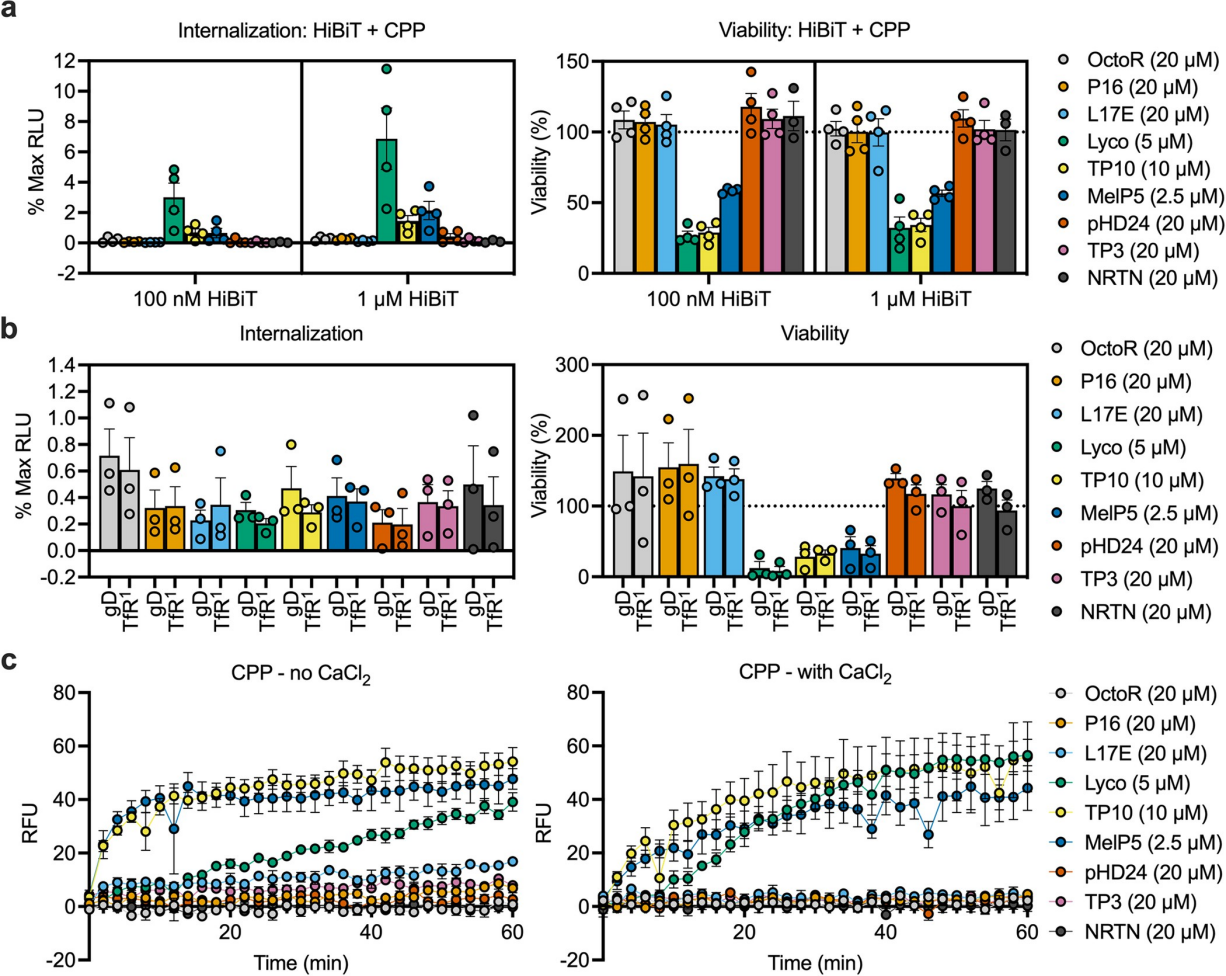
Delivery of cargoes by CPPs in *trans* relies on membrane damage. (a) Delivery of 100 nM and 1 μM HiBiT in *trans* by different CPPs after 30 minutes incubation. The concentration giving maximum delivery with submaximal toxicity is shown. with CPP concentrations indicated in the legend. Internalization is shown relative to cells incubated with HiBiT only and fully permeabilized with saponin. Viability is calculated relative to the respective DMSO + HiBiT control to account for toxicity due to DMSO addition. Bars show the mean, error bars the SEM, individual replicates are shown by symbols. *n =* 4, except for NRTN, where *n* = 3. (b) Delivery of 100 nM Fab-HiBiT fusions targeting either anti-gD or anti-TfR by different CPPs in *trans* after 30 min incubation. *n* = 3, bars show the mean, error bars show the SEM, individual replicates are shown as symbols. Viability and delivery are calculated as in (a), except that a Fab-HiBiT control is used. (c) Propidium iodide internalization into HeLa cells on incubation with different CPPs either in the absence (left) or presence (right) of calcium, to either inhibit or permit plasma membrane repair. Symbols show the mean of at least three replicates, error bars show the SEM. For all concentrations tested per peptide and experiment, see S5–S13 Figs. Data shown here is replicated in those figures for comparison.

If the delivery by CPPs is mediated mainly through plasma membrane damage, we would expect delivery both in *cis*, as well as in *trans*, since non-specific damage and pore formation should allow non-specific uptake of molecules from the media. We therefore assessed the ability of the CPP panel to mediate delivery of HiBiT peptide or Fab-HiBiT fusions in *trans* and measured PI fluorescence in the presence and absence of calcium to assess membrane damage. Results using the optimal CPP concentrations for each CPP are shown in Fig 5. For the full range of concentrations tested, per peptide, see S5–S13 Figs.

Surprisingly, only those CPPs previously identified as the most toxic, MelP5, TP10 and Lyco showed delivery of HiBiT peptide in *trans* (Fig 5A). When we compared the obtained luminescence to a fully permeabilized control, the maximum amount of HiBiT delivered was in the single digit percentage range, indicating that delivery is inefficient and limited by toxicity.

Delivery of 1 μM HiBiT appeared to be more efficient than delivery of 100 nM HiBiT, when comparing percentage delivery of the maximum relative light unit (RLU) observed. However, the relative delivery compared to the saponin control overestimates delivery of 1 μM HiBiT, due to saturation of the expressed LgBiT. We do not observe a 10-fold increase in the saponin permeabilized control when increasing the HiBiT concentration 10-fold, indicating that the concentration of LgBiT is limiting and that the percentage delivery for 1 μM HiBiT is an overestimate of the total delivery.

CPPs that showed no toxicity showed no delivery of HiBiT peptide. When we tested delivery of Fab-HiBiT fusions in *trans*, delivery was around an order of magnitude lower than for HiBiT peptide, indicating that the delivery mediated by membrane damage is impacted by cargo size, with the larger cargo showing much reduced delivery (Fig 5B). Toxicity correlated with membrane damage as shown by propidium iodide internalization (Fig 5C).

Interestingly, the CPPs OctoR, P16, L17E, TP3, and NRTN that had shown delivery and toxicity when fused to HiBiT were neither toxic, nor did they show delivery of HiBiT or Fab in *trans*. We also did not observe membrane damage from these CPPs in isolation, which contradicted the results we obtained with their respective HiBiT fusions (compare Figs 4B and 4D with 5C). This indicates that the HiBiT peptide itself alters the properties of the CPPs when evaluated as a fusion. Interestingly, HiBiT alone also did not mediate membrane damage (S4 Fig). We speculate that the positive charges within the HiBiT peptide increase membrane adsorption of the respective CPP HiBiT fusions peptides, increasing their local concentration on the membrane and increasing their potential for membrane disruption. This is consistent with previous studies showing that increased membrane adsorption leads to increased delivery of CPPs [35].

Despite these differences between the *trans* and *cis* properties of these CPPs, we again observed a marked correlation between delivery, toxicity and membrane damage mediated by these CPPs, indicating that their delivery potential is limited by toxicity. Additionally, when screening CPPs for delivery of a specific cargo, it appears that both the size and charge of the cargo can significantly alter the properties of the CPP.

## Discussion

Despite the strong interest and investment in CPPs over the past three decades, little progress has been made to transform them into efficient intracellular delivery modules for therapeutic use. It is poorly understood what limits this pursuit as previous engineering efforts and studies have mostly focused on improving or studying an individual CPP instead of class effects. A deeper understanding of these limitations has the potential to guide protein engineering strategies to improve future success rates. We have systematically and mechanistically compared a larger collection of previously described CPPs in a multi-dimensional experiment that investigates the correlation between peptide concentration, incubation time, cell fitness and cargo size. In addition, we dissected their function as isolated CPP from that as a fusion partner. Our results clearly demonstrate that cellular uptake is interconnected with cellular toxicity mediated by universal plasma membrane damage for the peptides studied. A fine balance is required to find the optimal concentration that allows delivery without causing severe and irreparable plasma membrane damage that ultimately leads to cell death. While the observation that CPPs can be toxic at higher concentrations is not novel, and has been extensively studied and reported [39], CPPs are frequently presented as being both high in internalization efficiency, as well as low in toxicity. Our current work shows that for the peptides tested, toxicity and internalization efficiency are correlated and that this toxicity puts an upper limit on internalization. As a consequence, only small amounts (<10%) of CPP and cargo can be delivered into the cells, explaining why in the past decades little improvement in delivery efficiency has been made.

While precise control over the CPP concentration is achievable in *in vitro* settings, it will be exceedingly difficult to control the systemic and local concentration *in vivo* as required, for example, for a therapeutic drug. This may limit the use of these CPPs in *in vivo* applications to amounts that are in the suboptimal delivery range. Additional hurdles that might limit translation of CPPs from *in vitro* work into the clinic is the observed reduction in potency when the CPPs are exposed to serum (S2 Fig and [38]). CPPs are typically tested in serum-free media, overestimating their true delivery potential *in vivo*.

Our work revealed that careful consideration of the cargo is also important as we have shown that the physicochemical nature and size of cargo influences uptake and toxicity. For example, plasma membrane damage was exacerbated by cargo with high positive net charge like our CPP-HiBiT fusions (Fig 4B and 4D). Larger sized cargo such as Fabs (Figs 4E, 5B) show reduced intracellular delivery efficiency. Interestingly, previous studies have also noted increased toxicity when cargo was conjugated to CPPs [40], similar to our observations that fusions between HiBiT and CPPs were more toxic and more membrane active than the CPPs alone. They noted that this effect appeared dependent on the cargo length, rather than its charge or composition. Additionally, fluorescent small molecule cargoes appear to sometimes enhance internalization and toxicity [40, 41]. This is of note since the majority of CPPs described were tested by assessing delivery of fluorescent dye-conjugated CPPs [8]. Fluorescent dyes are significantly smaller than therapeutically relevant protein cargo (S1 Table) and their delivery efficiency likely overestimates the delivery of larger cargo of therapeutic interest.

Studies with CPPs have in the past typically been conducted *in vitro* without the need to discriminate delivery between target and bystander cells. In contrast, *in vivo* settings require sparing non-target cells and tissues. Antibodies have been proposed as a solution to achieve the tissue specific delivery of CPPs, however, experimental data supporting this concept is scarce. Interestingly we did not observe improved delivery with a cell targeted Fab (anti-TfR^1^) compared to a non-targeted Fab (anti-gD), indicating a relatively high amount of non-specific delivery, since both Fabs showed background internalization when administered as a Fab alone. This unveils unexpected hurdles for the translation to a useful functional tool or therapeutic drug, since this non-specific targeting could cause both off-target toxicity, as well as reduced plasma concentrations due to non-specific clearance from the circulation. Expanding these studies to more receptors and cell types would be beneficial to clarify the generalizability of this observation.

Fab-CPP fusions that should deliver the molecule more efficiently to the endosome via receptor mediated endocytosis did not demonstrate an improvement in internalization over the CPP itself. Based on this lack of improvement in internalization, we hypothesize that the CPPs used in this study do not meaningfully allow the escape from the endosome. Instead, intracellular delivery is mainly achieved by non-specific association with and direct delivery through the plasma membrane. Further work is needed to gain more detailed insights, but more recent studies suggest that the positive charge of peptides and proteins is responsible for this non-specific association. For example Teo et al. showed that internalization correlates with plasma membrane association of the respective CPPs [35]. Additionally, a recent study [42] showed that depolarizing the plasma membrane to reduce surface association of CPPs abrogated internalization. It has been suggested that CPP association onto the cell surface triggers the formation of water pores with a 2–5 nm diameter in the membrane, allowing dyes such as PI and small molecules to enter [43, 44]. This is in line with our observations that plasma membrane damage and the resulting toxicity directly correlate with cell uptake of CPPs and CPP fusions, but uptake of larger Fab fragments is inefficient.

It is of note that the Fabs tested here are positively charged at neutral pH (~ +3 for anti-gD and ~ +8 for anti-TfR^1^ HiBiT fusion) and show a degree of background internalization even in the absence of CPPs. Positively charged proteins have previously been shown to have enhanced cell uptake [45], also in agreement with the hypothesis that non-specific plasma membrane association is a major contributor to uptake. However, while internalization of positively charged proteins is routinely observed, for example for transcription factors [46], DNA-binding antibodies [47], supercharged GFP [48] and other proteins [48, 49], we observed only low efficiencies for our positively charged Fabs. Maximum deliveries obtained, even in the presence of added CPP were <1% of total protein added. It seems unlikely that efficiencies sufficient for therapeutic effect of antibodies can be achieved through these methods. In agreement with this observation, Shin *et al*. [50] observed that, while an anti-Ras targeting internalizing antibody was able to suppress tumor growth to some extent in a mouse model, this required frequent dosing at very high doses, due to fast clearance, and inefficient internalization.

A potential area of use for these CPPs would be for the delivery of catalytic molecules, where small amounts delivered result in a large effect. Examples include the delivery of CRISPR-Cas9 [51], or other enzymes to affect gene function [11], or the delivery of enzymes to correct genetic defects [44]. In fact, studies showing an *in vivo* effect of CPP-mediated delivery of proteins often employ catalytic cargoes [43, 44, 52].

An additional avenue to improve CPP-mediated delivery would be provided by a better understanding of the trafficking mechanisms underlying CPP delivery to the cytoplasm. Recent studies have started to elucidate non-canonical entry pathways, employed by cationic CPPs as well as identifying molecular determinants for efficient delivery [53, 54].

In summary, the data presented in this work suggests that the CPPs tested here mediate non-specific delivery of cargo, through localized plasma membrane disruption. Delivery efficiency is affected by the nature of the cargo delivered and is limited in all cases by the toxicity resulting from interaction with the plasma membrane. We suggest that the canonical CPPs we evaluated are unlikely to be of use in a therapeutic setting for the delivery of non-catalytic cargoes, but that optimization efforts should be focused on increasing specificity, reducing toxicity and delivery of catalytic cargoes, to circumvent the need for high delivery efficiencies. Additionally, reports on the discovery of new CPPs should include a detailed investigation on membrane damage, membrane association, delivery mechanism, toxicity and delivery of different cargoes, preferably using standardized assays to allow comparison to previously identified CPPs.

## Supporting information

S1 Fig *In vitro* complementation assay of purified LgBiT with HiBiT fusion peptides. *In vitro* complementation of purified LgBiT protein by CPP HiBiT fusions *in vitro* after 30 min incubation. Assays were run three times, with different concentrations of HiBiT fusion. Each set of concentrations was run twice. Final assay concentration for LgBiT was 5 nM. To determine EC_50_, luminescence was normalized to the max value from the HiBiT control and fits analyzed using a nonlinear regression fit ([Agonist] vs. response, three parameters). EC_50_ values are shown in Table 2. Individual measurements are shown, lines show the non-linear fit.
(PDF)

S2 Fig Comparison of internalization for CPP HiBiT fusion peptides in serum-containing media and serum-free media. Serum-free data (HBSS) is the result of three independent experiments. Lines show the mean, error bars show the SEM. For serum-containing media (DMEM), the results are from two independent experiments. Lines show the mean, error bars show the SEM. Values are expressed as a fold increase over the HiBiT control. HBSS data is identical to the data shown in Fig 2 and is reproduced here for comparison to the DMEM experiments.
(PDF)

S3 Fig Comparison of toxicity for CPP HiBiT fusion peptides in serum-containing media and serum-free media. Serum-free experiments (HBSS) are the result of three independent experiments. Lines show the mean, error bars show the SEM. For serum-containing media (DMEM), the results are from two independent experiments. Lines show the mean, error bars show the SEM. Viability is calculated relative to the average of the cells-only control wells. HBSS data is identical to the data shown in Fig 2 and is reproduced here for comparison to the DMEM experiments.
(PDF)

S4 Fig Membrane damage by HiBiT peptide only. PI internalization using HiBiT peptide alone indicates no plasma membrane damage. The mean from five different experiments is shown, error bars show the SEM.
(PDF)

S5 Fig Delivery of different cargoes by the CPP OctoR. (a) Internalization of 100 nM (circles) or 1 μM (squares) HiBiT peptide, mediated by different concentrations of OctoR in *trans*. Internalization is expressed as a fold increase over no OctoR control wells, containing only HiBiT and a DMSO concentration equivalent to that in the OctoR added wells. Viability is calculated relative to this control to control for any effect of DMSO. (b) Membrane damage mediated by OctoR, measured by an increase in PI fluorescence over time, with a background DMSO only control subtracted from each OctoR concentration. (c, e) Internalization of HiBiT-OctoR fusions. Results are expressed relative to a HiBiT only control. (d, f) Membrane damage mediated by OctoR fusions to HiBiT, measured by PI fluorescence over time. Plots show DMSO background subtracted results. (g) Internalization of Fab fragments targeting gD (orange, circles) or transferrin receptor (blue, diamonds), mediated by different concentrations of OctoR in *trans*. All Fabs have a C-terminal HiBiT peptide on the HC, for detection in the luciferase complementation assay. Results are expressed relative to a no OctoR, Fab only control. (h) Internalization of Fab fragments targeting gD (orange, circles) or transferrin receptor (blue, diamonds) with OctoR fused to the C-terminus of the LC. All Fabs also contain a C-terminal HiBiT peptide on the HC. Results are expressed relative to a matching no OctoR control Fab. For (a, c, e, g) bars show the mean, error bars show the SEM. For (b, d and f) symbols show the mean and error bars show the SEM. Replicates were as follows: (a) *n* = 4, (b-h) *n* = 3. A subset of data from these Supplementary Figures is reproduced in Figs 3–5 in the main manuscript to show optimal concentrations for each CPP and side-by-side comparisons of the different CPPs.
(PDF)

S6 Fig Delivery of different cargoes by the CPP P16. (a) Internalization of 100 nM (circles) or 1 μM (squares) HiBiT peptide, mediated by different concentrations of P16 in trans. Internalization is expressed as a fold increase over no P16 control wells, containing only HiBiT and a DMSO concentration equivalent to that in the P16 added wells. Viability is calculated relative to this control to control for any effect of DMSO. (b) Membrane damage mediated by P16, measured by an increase in PI fluorescence over time, with a background DMSO only control subtracted from each P16 concentration. (c, e) Internalization of HiBiT-P16 fusions. Results are expressed relative to a HiBiT only control. (d, f) Membrane damage mediated by P16 fusions to HiBiT, measured by PI fluorescence over time. Plots show DMSO background subtracted results. (g) Internalization of Fab fragments targeting gD (orange, circles) or transferrin receptor (blue, diamonds), mediated by different concentrations of P16 in trans. All Fabs have a C-terminal HiBiT peptide on the HC, for detection in the luciferase complementation assay. Results are expressed relative to a no P16, Fab only control. (h) Internalization of Fab fragments targeting gD (orange, circles) or transferrin receptor (blue, diamonds) with P16 fused to the C-terminus of the LC. All Fabs also contain a C-terminal HiBiT peptide on the HC. Results are expressed relative to a matching no P16 control Fab. For (a, c, e, g) bars show the mean, error bars show the SEM. For (b, d and f) symbols show the mean and error bars show the SEM. Replicates were as follows: (a, b) n = 4, (c-h) n = 3. A subset of data from these Supplementary Figures is reproduced in Figs 3–5 in the main manuscript to show optimal concentrations for each CPP and side-by-side comparisons of the different CPPs.
(PDF)

S7 Fig Delivery of different cargoes by the CPP L17E. (a) Internalization of 100 nM (circles) or 1 μM (squares) HiBiT peptide, mediated by different concentrations of L17E in *trans*. Internalization is expressed as a fold increase over no L17E control wells, containing only HiBiT and a DMSO concentration equivalent to that in the L17E added wells. Viability is calculated relative to this control to control for any effect of DMSO. (b) Membrane damage mediated by L17E, measured by an increase in PI fluorescence over time, with a background DMSO only control subtracted from each L17E concentration. (c, e) Internalization of HiBiT-L17E fusions. Results are expressed relative to a HiBiT only control. (d, f) Membrane damage mediated by L17E fusions to HiBiT, measured by PI fluorescence over time. Plots show DMSO background subtracted results. (g) Internalization of Fab fragments targeting gD (orange, circles) or transferrin receptor (blue, diamonds), mediated by different concentrations of L17E in *trans*. All Fabs have a C-terminal HiBiT peptide on the HC, for detection in the luciferase complementation assay. Results are expressed relative to a no L17E, Fab only control. (h) Internalization of Fab fragments targeting gD (orange, circles) or transferrin receptor (blue, diamonds) with L17E fused to the C-terminus of the LC. All Fabs also contain a C-terminal HiBiT peptide on the HC. Results are expressed relative to a matching no L17E control Fab. For (a, c, e, g) bars show the mean, error bars show the SEM. For (b, d and f) symbols show the mean and error bars show the SEM. Replicates were as follows: (a, d, f) *n* = 4, (b, c, e, g, h) *n* = 3. A subset of data from these Supplementary Figures is reproduced in Figs 3–5 in the main manuscript to show optimal concentrations for each CPP and side-by-side comparisons of the different CPPs.
(PDF)

S8 Fig Delivery of different cargoes by the CPP Lyco. (a) Internalization of 100 nM (circles) or 1 μM (squares) HiBiT peptide, mediated by different concentrations of Lyco in *trans*. Internalization is expressed as a fold increase over no Lyco control wells, containing only HiBiT and a DMSO concentration equivalent to that in the Lyco added wells. Viability is calculated relative to this control to control for any effect of DMSO. (b) Membrane damage mediated by Lyco, measured by an increase in PI fluorescence over time, with a background DMSO only control subtracted from each Lyco concentration. (c, e) Internalization of HiBiT-Lyco fusions. Results are expressed relative to a HiBiT only control. (d, f) Membrane damage mediated by Lyco fusions to HiBiT, measured by PI fluorescence over time. Plots show DMSO background subtracted results. (g) Internalization of Fab fragments targeting gD (orange, circles) or transferrin receptor (blue, diamonds), mediated by different concentrations of Lyco in *trans*. All Fabs have a C-terminal HiBiT peptide on the HC, for detection in the luciferase complementation assay. Results are expressed relative to a no Lyco, Fab only control. (h) Internalization of Fab fragments targeting gD (orange, circles) or transferrin receptor (blue, diamonds) with Lyco fused to the C-terminus of the LC. All Fabs also contain a C-terminal HiBiT peptide on the HC. Results are expressed relative to a matching no Lyco control Fab. For (a, c, e, g) bars show the mean, error bars show the SEM. For (b, d and f) symbols show the mean and error bars show the SEM. Replicates were as follows: (a, b) *n* = 4, (c-h) *n* = 3. A subset of data from these Supplementary Figures is reproduced in Figs 3–5 in the main manuscript to show optimal concentrations for each CPP and side-by-side comparisons of the different CPPs.
(PDF)

S9 Fig Delivery of different cargoes by the CPP TP10. (a) Internalization of 100 nM (circles) or 1 μM (squares) HiBiT peptide, mediated by different concentrations of TP10 in *trans*. Internalization is expressed as a fold increase over no TP10 control wells, containing only HiBiT and a DMSO concentration equivalent to that in the TP10 added wells. Viability is calculated relative to this control to control for any effect of DMSO. (b) Membrane damage mediated by TP10, measured by an increase in PI fluorescence over time, with a background DMSO only control subtracted from each TP10 concentration. (c, e) Internalization of HiBiT-TP10 fusions. Results are expressed relative to a HiBiT only control. (d, f) Membrane damage mediated by TP10 fusions to HiBiT, measured by PI fluorescence over time. Plots show DMSO background subtracted results. (g) Internalization of Fab fragments targeting gD (orange, circles) or transferrin receptor (blue, diamonds), mediated by different concentrations of TP10 in *trans*. All Fabs have a C-terminal HiBiT peptide on the HC, for detection in the luciferase complementation assay. Results are expressed relative to a no TP10, Fab only control. (h) Internalization of Fab fragments targeting gD (orange, circles) or transferrin receptor (blue, diamonds) with TP10 fused to the C-terminus of the LC. All Fabs also contain a C-terminal HiBiT peptide on the HC. Results are expressed relative to a matching no TP10 control Fab. For (a, c, e, g) bars show the mean, error bars show the SEM. For (b, d and f) symbols show the mean and error bars show the SEM. Replicates were as follows: (a, b) *n* = 4, (c-h) *n* = 3. A subset of data from these Supplementary Figures is reproduced in Figs 3–5 in the main manuscript to show optimal concentrations for each CPP and side-by-side comparisons of the different CPPs.
(PDF)

S10 Fig Delivery of different cargoes by the CPP MelP5. (a) Internalization of 100 nM (circles) or 1 μM (squares) HiBiT peptide, mediated by different concentrations of MelP5 in *trans*. Internalization is expressed as a fold increase over no MelP5 control wells, containing only HiBiT and a DMSO concentration equivalent to that in the MelP5 added wells. Viability is calculated relative to this control to control for any effect of DMSO. (b) Membrane damage mediated by MelP5, measured by an increase in PI fluorescence over time, with a background DMSO only control subtracted from each MelP5 concentration. (c, e) Internalization of HiBiT-MelP5 fusions. Results are expressed relative to a HiBiT only control. (d, f) Membrane damage mediated by MelP5 fusions to HiBiT, measured by PI fluorescence over time. Plots show DMSO background subtracted results. (g) Internalization of Fab fragments targeting gD (orange, circles) or transferrin receptor (blue, diamonds), mediated by different concentrations of MelP5 in *trans*. All Fabs have a C-terminal HiBiT peptide on the HC, for detection in the luciferase complementation assay. Results are expressed relative to a no MelP5, Fab only control. (h) Internalization of Fab fragments targeting gD (orange, circles) or transferrin receptor (blue, diamonds) with MelP5 fused to the C-terminus of the LC. All Fabs also contain a C-terminal HiBiT peptide on the HC. Results are expressed relative to a matching no MelP5 control Fab. For (a, c, e, g) bars show the mean, error bars show the SEM. For (b, d and f) symbols show the mean and error bars show the SEM. Replicates were as follows: (a) *n* = 4, (b-h) *n* = 3. A subset of data from these Supplementary Figures is reproduced in Figs 3–5 in the main manuscript to show optimal concentrations for each CPP and side-by-side comparisons of the different CPPs.
(PDF)

S11 Fig Delivery of different cargoes by the CPP pHD24. (a) Internalization of 100 nM (circles) or 1 μM (squares) HiBiT peptide, mediated by different concentrations of pHD24 in *trans*. Internalization is expressed as a fold increase over no pHD24 control wells, containing only HiBiT and a DMSO concentration equivalent to that in the pHD24 added wells. Viability is calculated relative to this control to control for any effect of DMSO. (b) Membrane damage mediated by pHD24, measured by an increase in PI fluorescence over time, with a background DMSO only control subtracted from each pHD24 concentration. (c, e) Internalization of HiBiT-pHD24 fusions. Results are expressed relative to a HiBiT only control. (d, f) Membrane damage mediated by pHD24 fusions to HiBiT, measured by PI fluorescence over time. Plots show DMSO background subtracted results. (g) Internalization of Fab fragments targeting gD (orange, circles) or transferrin receptor (blue, diamonds), mediated by different concentrations of pHD24 in *trans*. All Fabs have a C-terminal HiBiT peptide on the HC, for detection in the luciferase complementation assay. Results are expressed relative to a no pHD24, Fab only control. (h) Internalization of Fab fragments targeting gD (orange, circles) or transferrin receptor (blue, diamonds) with pHD24 fused to the C-terminus of the LC. All Fabs also contain a C-terminal HiBiT peptide on the HC. Results are expressed relative to a matching no pHD24 control Fab. For (a, c, e, g) bars show the mean, error bars show the SEM. For (b, d and f) symbols show the mean and error bars show the SEM. Replicates were as follows: (a) *n* = 4, (b-h) *n* = 3. A subset of data from these Supplementary Figures is reproduced in Figs 3–5 in the main manuscript to show optimal concentrations for each CPP and side-by-side comparisons of the different CPPs.
(PDF)

S12 Fig Delivery of different cargoes by the CPP TP3. (a) Internalization of 100 nM (circles) or 1 μM (squares) HiBiT peptide, mediated by different concentrations of TP3 in *trans*. Internalization is expressed as a fold increase over no TP3 control wells, containing only HiBiT and a DMSO concentration equivalent to that in the TP3 added wells. Viability is calculated relative to this control to control for any effect of DMSO. (b) Membrane damage mediated by TP3, measured by an increase in PI fluorescence over time, with a background DMSO only control subtracted from each TP3 concentration. (c, e) Internalization of HiBiT-TP3 fusions. Results are expressed relative to a HiBiT only control. (d, f) Membrane damage mediated by TP3 fusions to HiBiT, measured by PI fluorescence over time. Plots show DMSO background subtracted results. (g) Internalization of Fab fragments targeting gD (orange, circles) or transferrin receptor (blue, diamonds), mediated by different concentrations of TP3 in *trans*. All Fabs have a C-terminal HiBiT peptide on the HC, for detection in the luciferase complementation assay. Results are expressed relative to a no TP3, Fab only control. (h) Internalization of Fab fragments targeting gD (orange, circles) or transferrin receptor (blue, diamonds) with TP3 fused to the C-terminus of the LC. All Fabs also contain a C-terminal HiBiT peptide on the HC. Results are expressed relative to a matching no TP3 control Fab. For (a, c, e, g) bars show the mean, error bars show the SEM. For (b, d and f) symbols show the mean and error bars show the SEM. Replicates were as follows: (a, b) *n* = 4, (c-h) *n* = 3. A subset of data from these Supplementary Figures is reproduced in Figs 3–5 in the main manuscript to show optimal concentrations for each CPP and side-by-side comparisons of the different CPPs.
(PDF)

S13 Fig Delivery of different cargoes by the CPP NRTN. (a) Internalization of 100 nM (circles) or 1 μM (squares) HiBiT peptide, mediated by different concentrations of NRTN in *trans*. Internalization is expressed as a fold increase over no NRTN control wells, containing only HiBiT and a DMSO concentration equivalent to that in the NRTN added wells. Viability is calculated relative to this control to control for any effect of DMSO. (b) Membrane damage mediated by NRTN, measured by an increase in PI fluorescence over time, with a background DMSO only control subtracted from each NRTN concentration. (c, e) Internalization of HiBiT-NRTN fusions. Results are expressed relative to a HiBiT only control. (d, f) Membrane damage mediated by NRTN fusions to HiBiT, measured by PI fluorescence over time. Plots show DMSO background subtracted results. (g) Internalization of Fab fragments targeting gD (orange, circles) or transferrin receptor (blue, diamonds), mediated by different concentrations of NRTN in *trans*. All Fabs have a C-terminal HiBiT peptide on the HC, for detection in the luciferase complementation assay. Results are expressed relative to a no NRTN, Fab only control. (h) Internalization of Fab fragments targeting gD (orange, circles) or transferrin receptor (blue, diamonds) with NRTN fused to the C-terminus of the LC. All Fabs also contain a C-terminal HiBiT peptide on the HC. Results are expressed relative to a matching no NRTN control Fab. For (a, c, e, g) bars show the mean, error bars show the SEM. For (b, d and f) symbols show the mean and error bars show the SEM. Replicates were as follows: (a) *n* = 4, (b-h) *n* = 3. A subset of data from these Supplementary Figures is reproduced in Figs 3–5 in the main manuscript to show optimal concentrations for each CPP and side-by-side comparisons of the different CPPs.
(PDF)

S14 Fig Final purification yields for Fab-CPP fusions in different orientations. All yields are from 30 ml expressions, purified using a single-step affinity purification using CH1-XL resin. Individual replicates are shown. Bars show either a single replicate, or the mean of multiple replicates where available. For multiple replicates, error bars show the SEM.
(PDF)

S15 Fig Comparison of internalization of N- and C-terminal CPP fusions to anti-gD Fab fragments. Raw luminescence values for internalization are plotted on the left axis, raw luminescence values for viability (CTG) are plotted on the right axis. *n* = 2, bars show the mean. Fabs with the CPP on the LC N-terminus are shown in red, LC C-terminus in blue.
(PDF)

S16 Fig Comparison of internalization of N- and C-terminal CPP fusions to anti-TfR^1^ Fab fragments. Raw luminescence values for internalization are plotted on the left axis, raw luminescence values for viability (CTG) are plotted on the right axis. *n* = 2, bars show the mean. Fabs with the CPP on the LC N-terminus are shown in red, LC C-terminus in blue.
(PDF)

S1 Table Size comparison of broadly utilized cargo for assessing intracellular delivery of CPPs. (PDF)

S1 Data (XLSX)

S1 File (PDF)
