# Supplementary figures and images for: Plasma membrane damage limits cytoplasmic delivery by conventional cell penetrating peptides
Source: PLoS One. 2024 Sep 3;19(9):e0305848. doi: 10.1371/journal.pone.0305848 (PMC11371239; doi:10.1371/journal.pone.0305848)

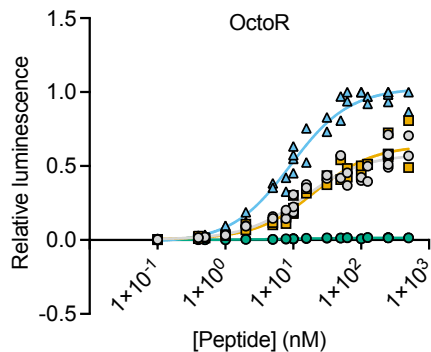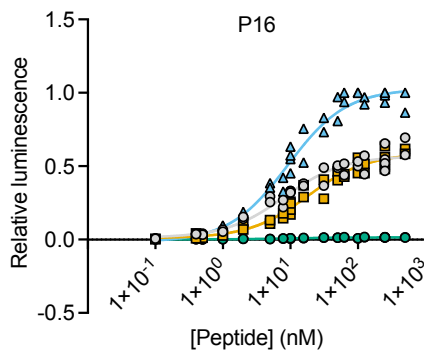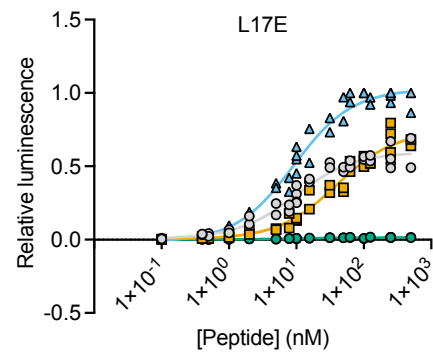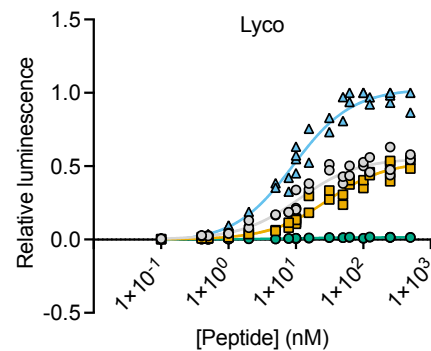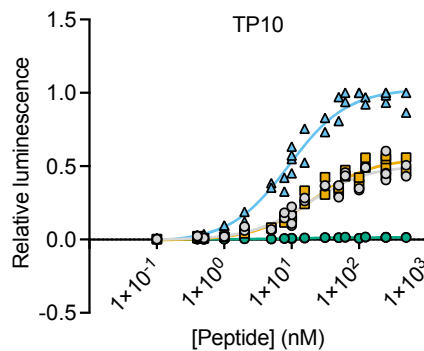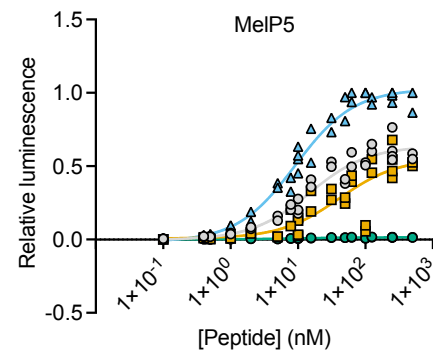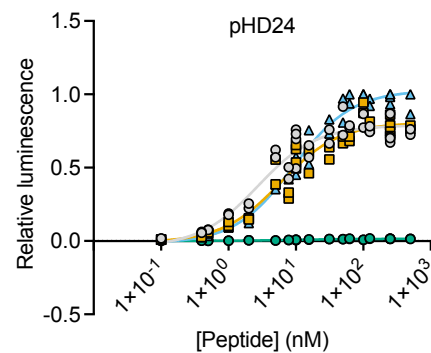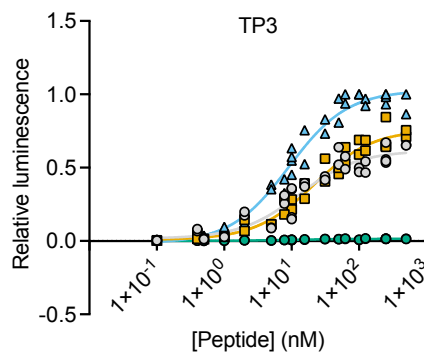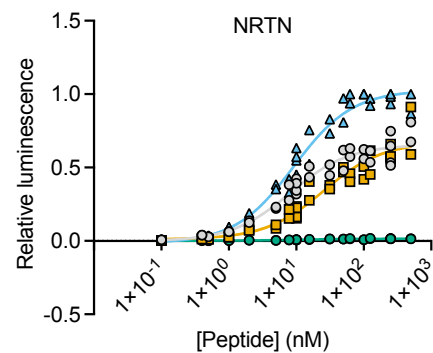

○ CPP-HiBiT

□ HiBiT-CPP

△ HiBiT

● DMSO

Supplement: S1 Fig — In vitro complementation of purified LgBiT protein by CPP HiBiT fusions in vitro after 30 min incubation. Assays were run three times, with different concentrations of HiBiT fusion. Each set of concentrations was run twice. Final assay concentration for LgBiT was 5 nM. To determine EC50, luminescence was normalized to the max value from the HiBiT control and fits analyzed using a nonlinear regression fit ([Agonist] vs. response, three parameters). EC50 values are shown in Table 2. Individual measurements are shown, lines show the non-linear fit. (PDF) [file pone.0305848.s001.pdf]

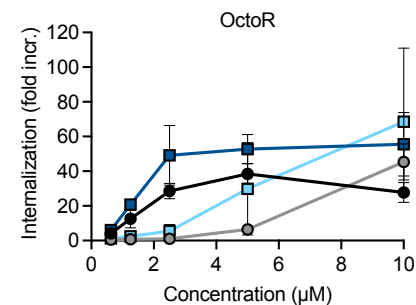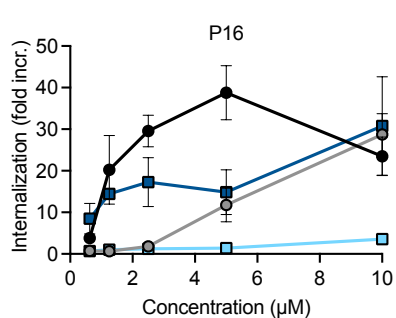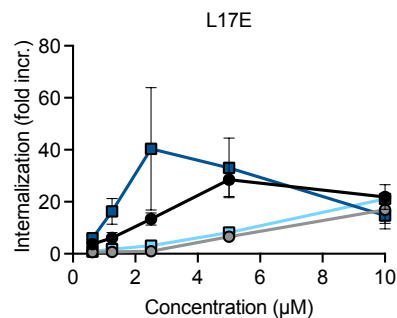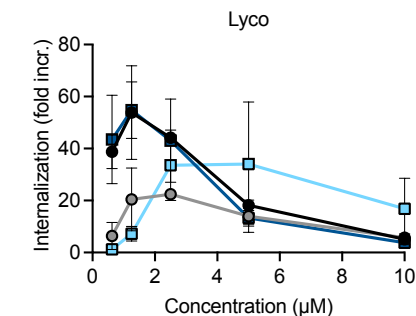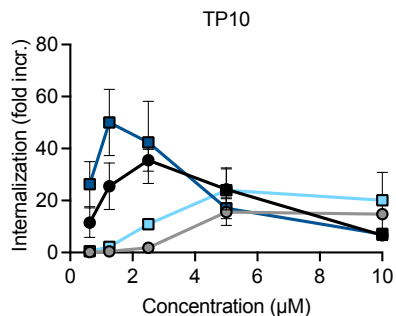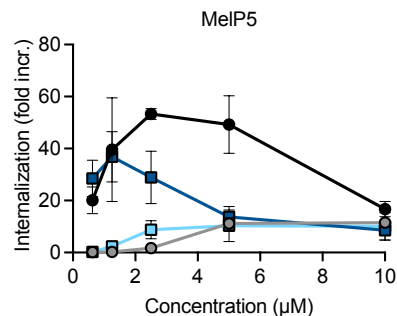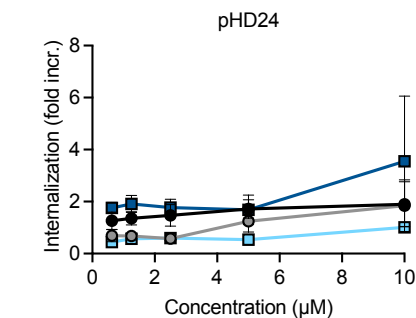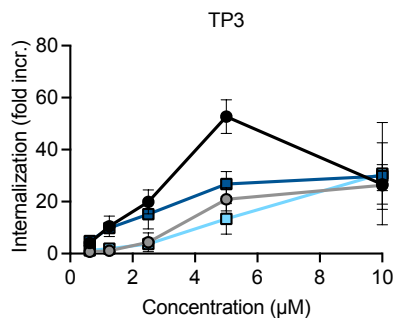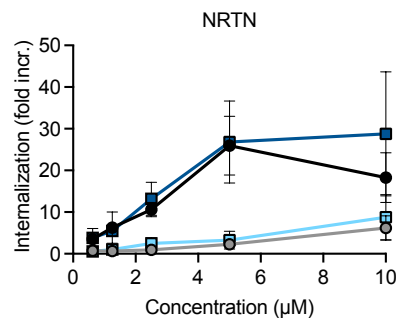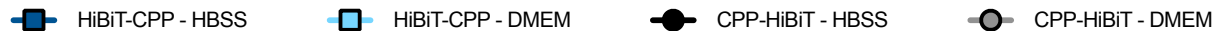

Supplement: S2 Fig — Serum-free data (HBSS) is the result of three independent experiments. Lines show the mean, error bars show the SEM. For serum-containing media (DMEM), the results are from two independent experiments. Lines show the mean, error bars show the SEM. Values are expressed as a fold increase over the HiBiT control. HBSS data is identical to the data shown in Fig 2 and is reproduced here for comparison to the DMEM experiments. (PDF) [file pone.0305848.s002.pdf]

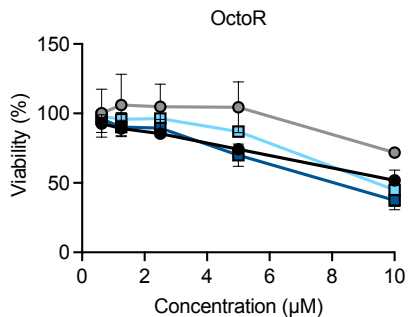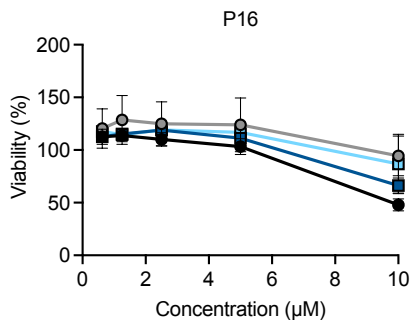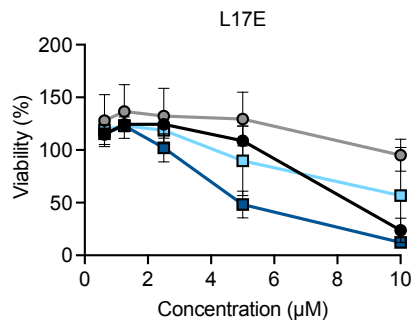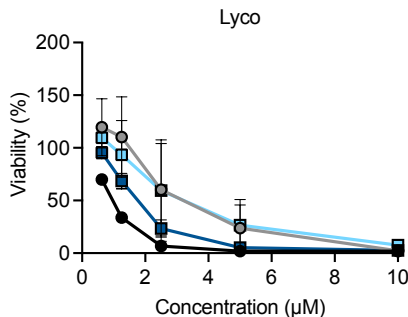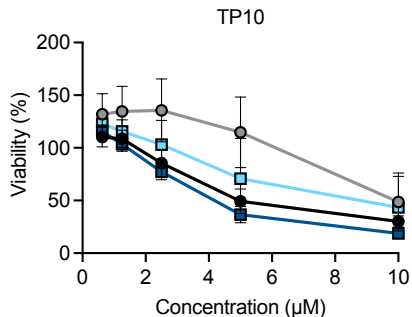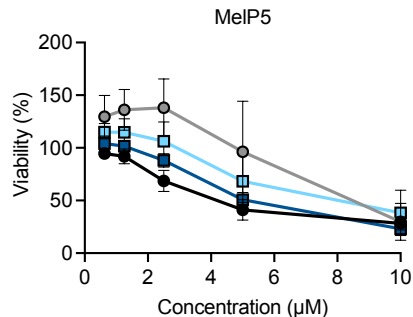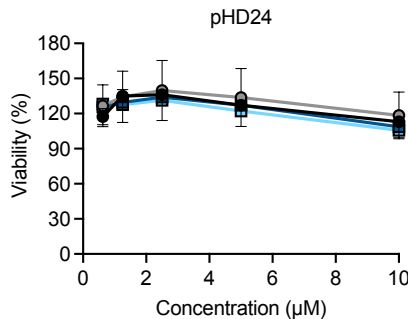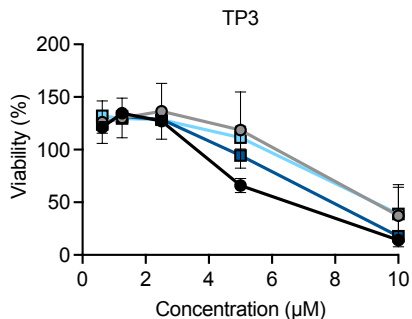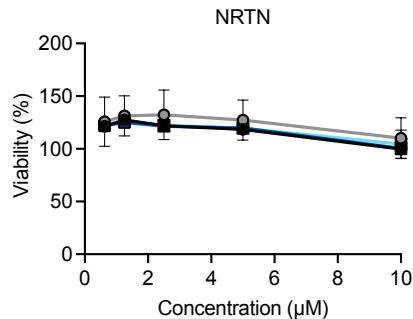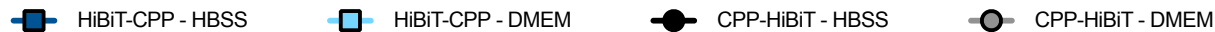

Supplement: S3 Fig — Serum-free experiments (HBSS) are the result of three independent experiments. Lines show the mean, error bars show the SEM. For serum-containing media (DMEM), the results are from two independent experiments. Lines show the mean, error bars show the SEM. Viability is calculated relative to the average of the cells-only control wells. HBSS data is identical to the data shown in Fig 2 and is reproduced here for comparison to the DMEM experiments. (PDF) [file pone.0305848.s003.pdf]

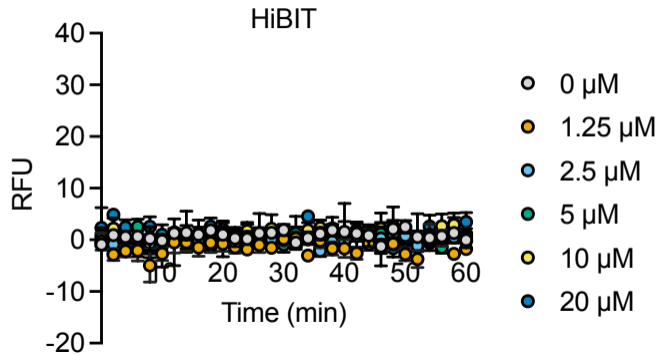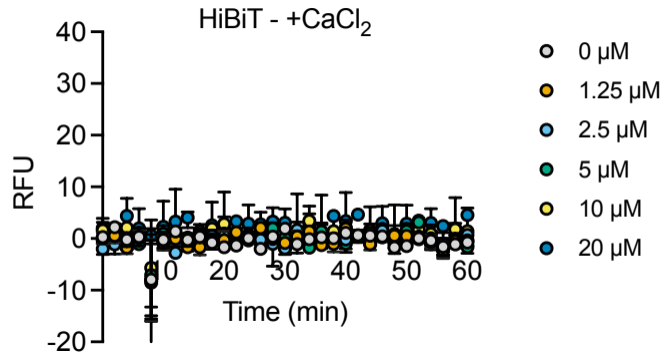

Supplement: S4 Fig — PI internalization using HiBiT peptide alone indicates no plasma membrane damage. The mean from five different experiments is shown, error bars show the SEM. (PDF) [file pone.0305848.s004.pdf]

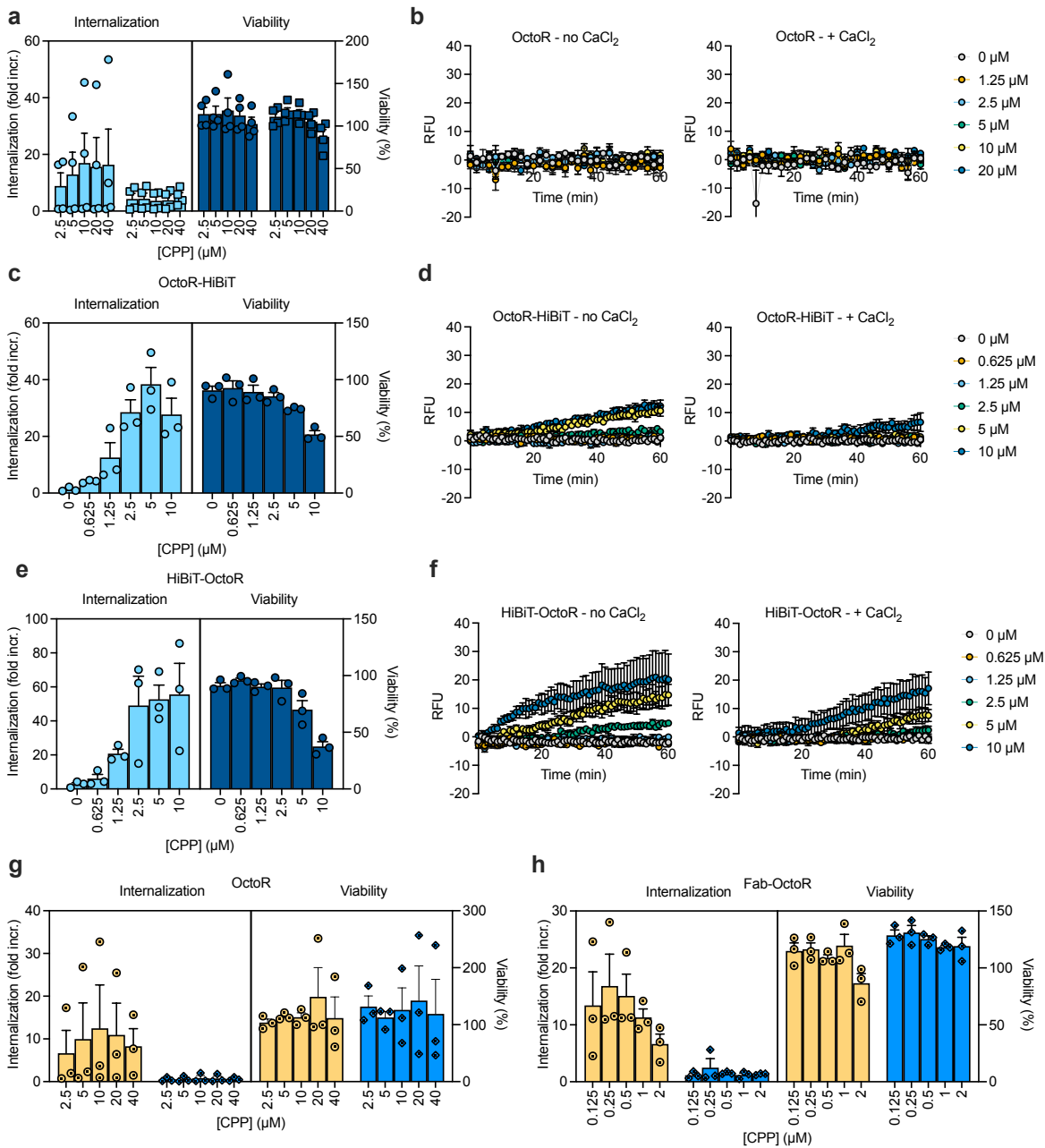

Supplement: S5 Fig — (a) Internalization of 100 nM (circles) or 1 μM (squares) HiBiT peptide, mediated by different concentrations of OctoR in trans. Internalization is expressed as a fold increase over no OctoR control wells, containing only HiBiT and a DMSO concentration equivalent to that in the OctoR added wells. Viability is calculated relative to this control to control for any effect of DMSO. (b) Membrane damage mediated by OctoR, measured by an increase in PI fluorescence over time, with a background DMSO only control subtracted from each OctoR concentration. (c, e) Internalization of HiBiT-OctoR fusions. Results are expressed relative to a HiBiT only control. (d, f) Membrane damage mediated by OctoR fusions to HiBiT, measured by PI fluorescence over time. Plots show DMSO background subtracted results. (g) Internalization of Fab fragments targeting gD (orange, circles) or transferrin receptor (blue, diamonds), mediated by different concentrations of OctoR in trans. All Fabs have a C-terminal HiBiT peptide on the HC, for detection in the luciferase complementation assay. Results are expressed relative to a no OctoR, Fab only control. (h) Internalization of Fab fragments targeting gD (orange, circles) or transferrin receptor (blue, diamonds) with OctoR fused to the C-terminus of the LC. All Fabs also contain a C-terminal HiBiT peptide on the HC. Results are expressed relative to a matching no OctoR control Fab. For (a, c, e, g) bars show the mean, error bars show the SEM. For (b, d and f) symbols show the mean and error bars show the SEM. Replicates were as follows: (a) n = 4, (b-h) n = 3. A subset of data from these Supplementary Figures is reproduced in Figs 3–5 in the main manuscript to show optimal concentrations for each CPP and side-by-side comparisons of the different CPPs. (PDF) [file pone.0305848.s005.pdf]

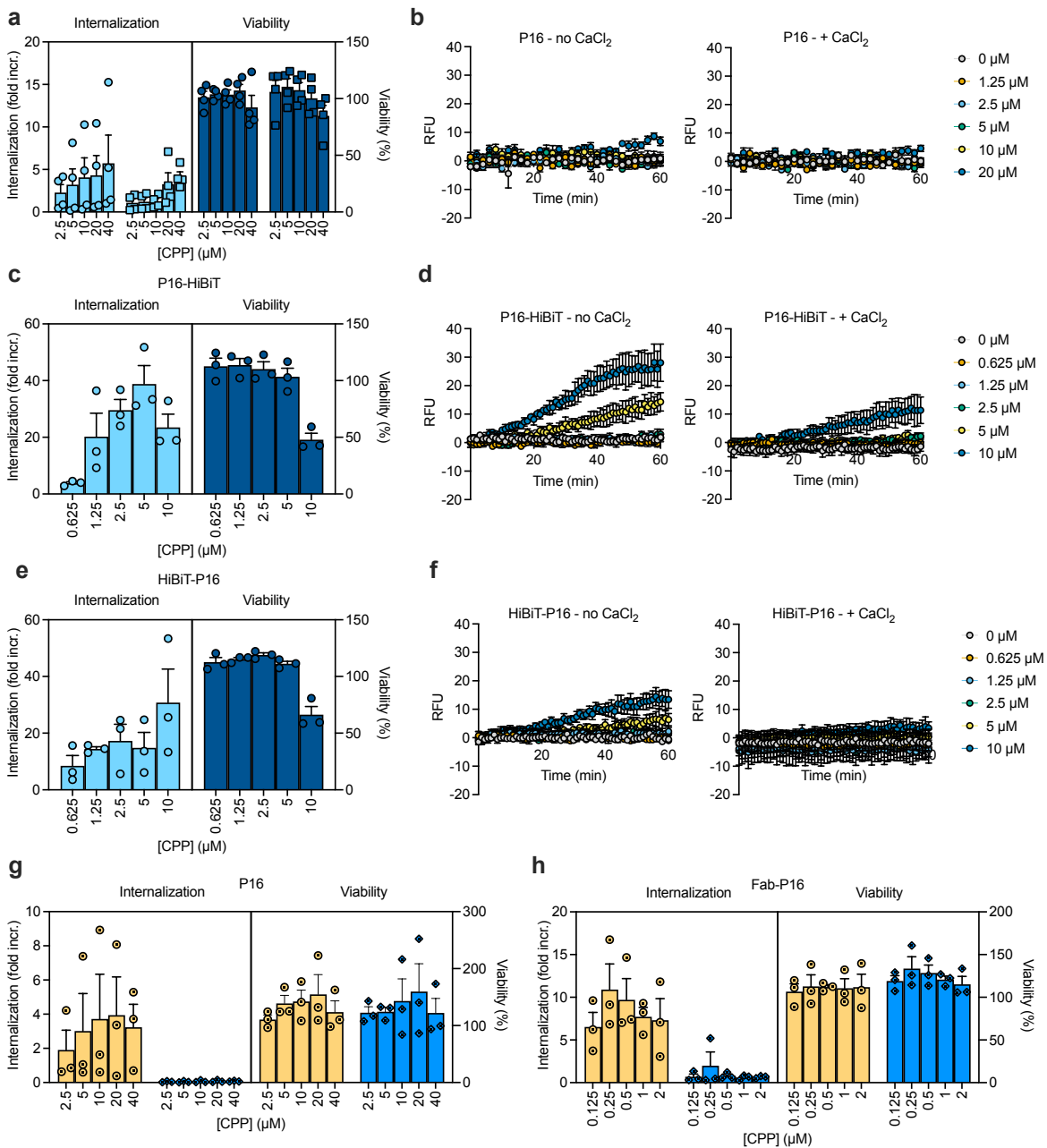

Supplement: S6 Fig — (a) Internalization of 100 nM (circles) or 1 μM (squares) HiBiT peptide, mediated by different concentrations of P16 in trans. Internalization is expressed as a fold increase over no P16 control wells, containing only HiBiT and a DMSO concentration equivalent to that in the P16 added wells. Viability is calculated relative to this control to control for any effect of DMSO. (b) Membrane damage mediated by P16, measured by an increase in PI fluorescence over time, with a background DMSO only control subtracted from each P16 concentration. (c, e) Internalization of HiBiT-P16 fusions. Results are expressed relative to a HiBiT only control. (d, f) Membrane damage mediated by P16 fusions to HiBiT, measured by PI fluorescence over time. Plots show DMSO background subtracted results. (g) Internalization of Fab fragments targeting gD (orange, circles) or transferrin receptor (blue, diamonds), mediated by different concentrations of P16 in trans. All Fabs have a C-terminal HiBiT peptide on the HC, for detection in the luciferase complementation assay. Results are expressed relative to a no P16, Fab only control. (h) Internalization of Fab fragments targeting gD (orange, circles) or transferrin receptor (blue, diamonds) with P16 fused to the C-terminus of the LC. All Fabs also contain a C-terminal HiBiT peptide on the HC. Results are expressed relative to a matching no P16 control Fab. For (a, c, e, g) bars show the mean, error bars show the SEM. For (b, d and f) symbols show the mean and error bars show the SEM. Replicates were as follows: (a, b) n = 4, (c-h) n = 3. A subset of data from these Supplementary Figures is reproduced in Figs 3–5 in the main manuscript to show optimal concentrations for each CPP and side-by-side comparisons of the different CPPs. (PDF) [file pone.0305848.s006.pdf]

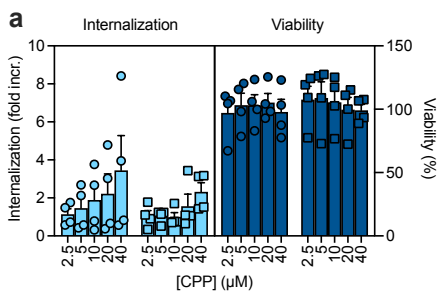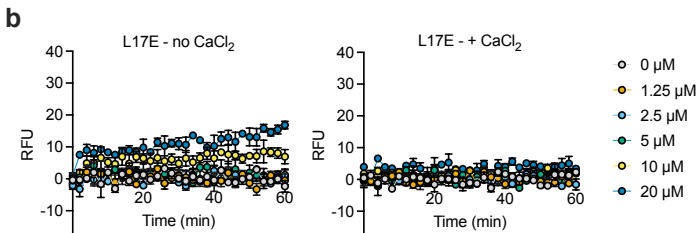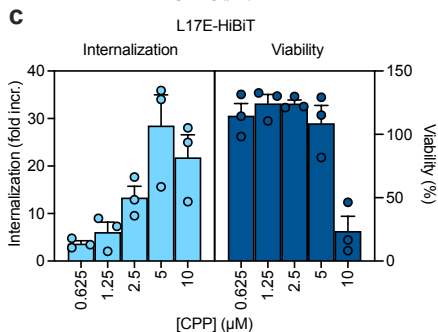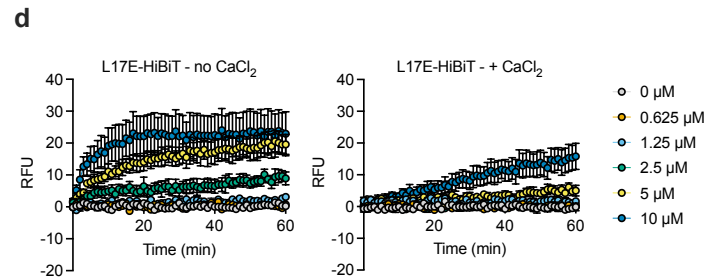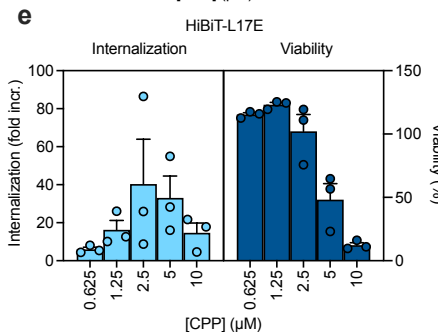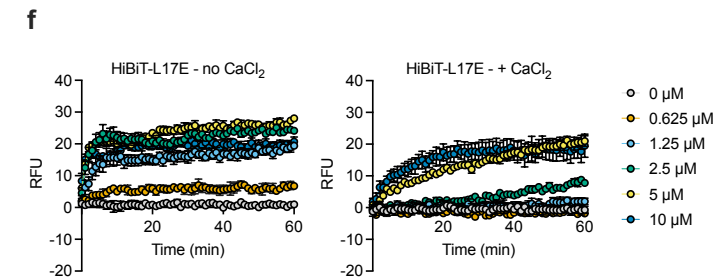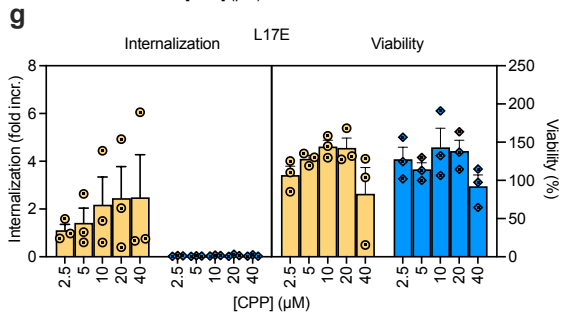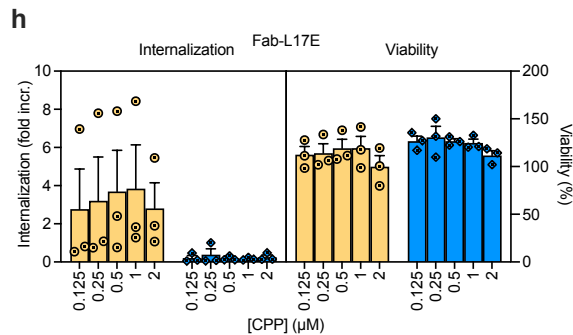

Supplement: S7 Fig — (a) Internalization of 100 nM (circles) or 1 μM (squares) HiBiT peptide, mediated by different concentrations of L17E in trans. Internalization is expressed as a fold increase over no L17E control wells, containing only HiBiT and a DMSO concentration equivalent to that in the L17E added wells. Viability is calculated relative to this control to control for any effect of DMSO. (b) Membrane damage mediated by L17E, measured by an increase in PI fluorescence over time, with a background DMSO only control subtracted from each L17E concentration. (c, e) Internalization of HiBiT-L17E fusions. Results are expressed relative to a HiBiT only control. (d, f) Membrane damage mediated by L17E fusions to HiBiT, measured by PI fluorescence over time. Plots show DMSO background subtracted results. (g) Internalization of Fab fragments targeting gD (orange, circles) or transferrin receptor (blue, diamonds), mediated by different concentrations of L17E in trans. All Fabs have a C-terminal HiBiT peptide on the HC, for detection in the luciferase complementation assay. Results are expressed relative to a no L17E, Fab only control. (h) Internalization of Fab fragments targeting gD (orange, circles) or transferrin receptor (blue, diamonds) with L17E fused to the C-terminus of the LC. All Fabs also contain a C-terminal HiBiT peptide on the HC. Results are expressed relative to a matching no L17E control Fab. For (a, c, e, g) bars show the mean, error bars show the SEM. For (b, d and f) symbols show the mean and error bars show the SEM. Replicates were as follows: (a, d, f) n = 4, (b, c, e, g, h) n = 3. A subset of data from these Supplementary Figures is reproduced in Figs 3–5 in the main manuscript to show optimal concentrations for each CPP and side-by-side comparisons of the different CPPs. (PDF) [file pone.0305848.s007.pdf]

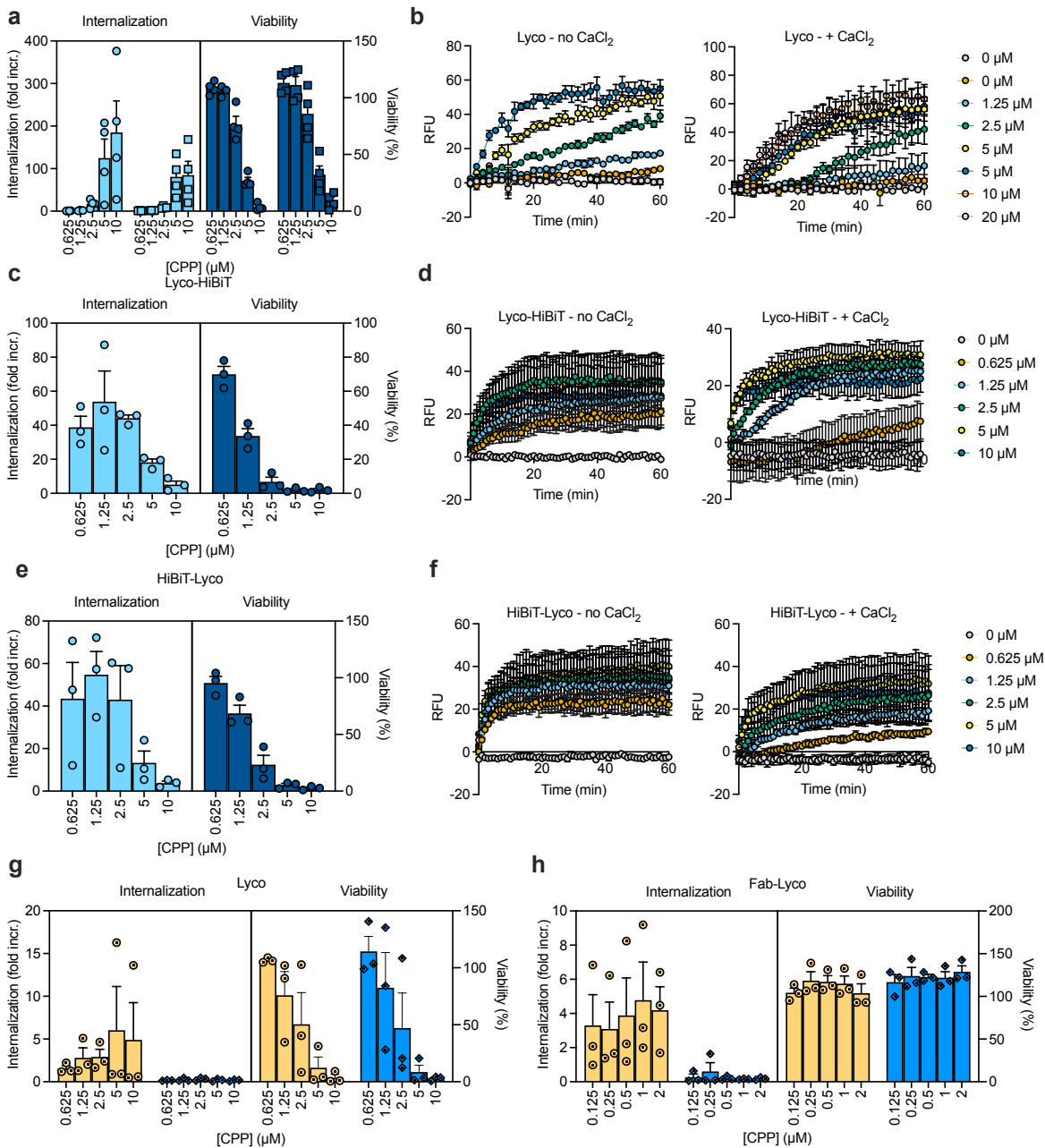

Supplement: S8 Fig — (a) Internalization of 100 nM (circles) or 1 μM (squares) HiBiT peptide, mediated by different concentrations of Lyco in trans. Internalization is expressed as a fold increase over no Lyco control wells, containing only HiBiT and a DMSO concentration equivalent to that in the Lyco added wells. Viability is calculated relative to this control to control for any effect of DMSO. (b) Membrane damage mediated by Lyco, measured by an increase in PI fluorescence over time, with a background DMSO only control subtracted from each Lyco concentration. (c, e) Internalization of HiBiT-Lyco fusions. Results are expressed relative to a HiBiT only control. (d, f) Membrane damage mediated by Lyco fusions to HiBiT, measured by PI fluorescence over time. Plots show DMSO background subtracted results. (g) Internalization of Fab fragments targeting gD (orange, circles) or transferrin receptor (blue, diamonds), mediated by different concentrations of Lyco in trans. All Fabs have a C-terminal HiBiT peptide on the HC, for detection in the luciferase complementation assay. Results are expressed relative to a no Lyco, Fab only control. (h) Internalization of Fab fragments targeting gD (orange, circles) or transferrin receptor (blue, diamonds) with Lyco fused to the C-terminus of the LC. All Fabs also contain a C-terminal HiBiT peptide on the HC. Results are expressed relative to a matching no Lyco control Fab. For (a, c, e, g) bars show the mean, error bars show the SEM. For (b, d and f) symbols show the mean and error bars show the SEM. Replicates were as follows: (a, b) n = 4, (c-h) n = 3. A subset of data from these Supplementary Figures is reproduced in Figs 3–5 in the main manuscript to show optimal concentrations for each CPP and side-by-side comparisons of the different CPPs. (PDF) [file pone.0305848.s008.pdf]

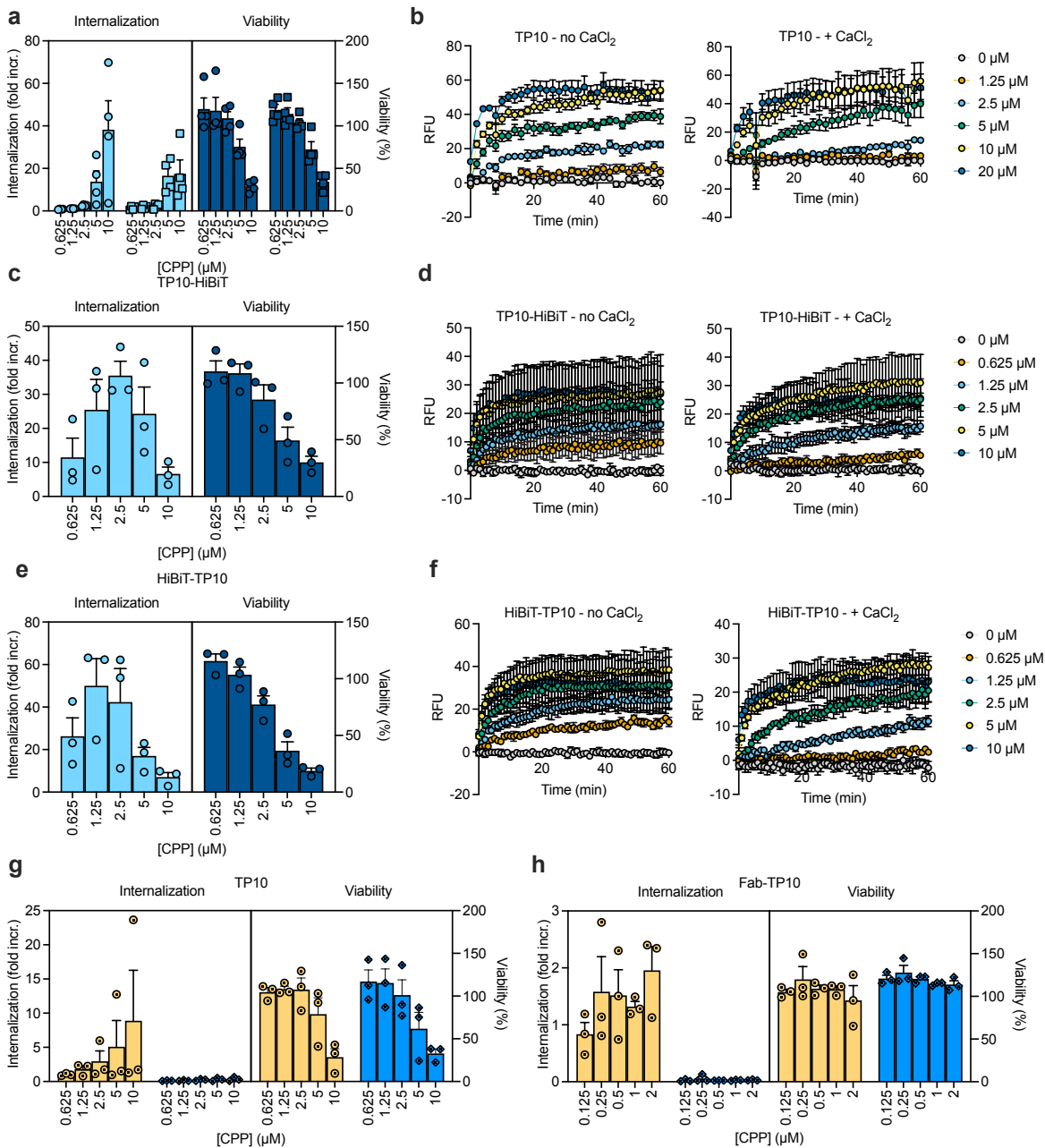

Supplement: S9 Fig — (a) Internalization of 100 nM (circles) or 1 μM (squares) HiBiT peptide, mediated by different concentrations of TP10 in trans. Internalization is expressed as a fold increase over no TP10 control wells, containing only HiBiT and a DMSO concentration equivalent to that in the TP10 added wells. Viability is calculated relative to this control to control for any effect of DMSO. (b) Membrane damage mediated by TP10, measured by an increase in PI fluorescence over time, with a background DMSO only control subtracted from each TP10 concentration. (c, e) Internalization of HiBiT-TP10 fusions. Results are expressed relative to a HiBiT only control. (d, f) Membrane damage mediated by TP10 fusions to HiBiT, measured by PI fluorescence over time. Plots show DMSO background subtracted results. (g) Internalization of Fab fragments targeting gD (orange, circles) or transferrin receptor (blue, diamonds), mediated by different concentrations of TP10 in trans. All Fabs have a C-terminal HiBiT peptide on the HC, for detection in the luciferase complementation assay. Results are expressed relative to a no TP10, Fab only control. (h) Internalization of Fab fragments targeting gD (orange, circles) or transferrin receptor (blue, diamonds) with TP10 fused to the C-terminus of the LC. All Fabs also contain a C-terminal HiBiT peptide on the HC. Results are expressed relative to a matching no TP10 control Fab. For (a, c, e, g) bars show the mean, error bars show the SEM. For (b, d and f) symbols show the mean and error bars show the SEM. Replicates were as follows: (a, b) n = 4, (c-h) n = 3. A subset of data from these Supplementary Figures is reproduced in Figs 3–5 in the main manuscript to show optimal concentrations for each CPP and side-by-side comparisons of the different CPPs. (PDF) [file pone.0305848.s009.pdf]

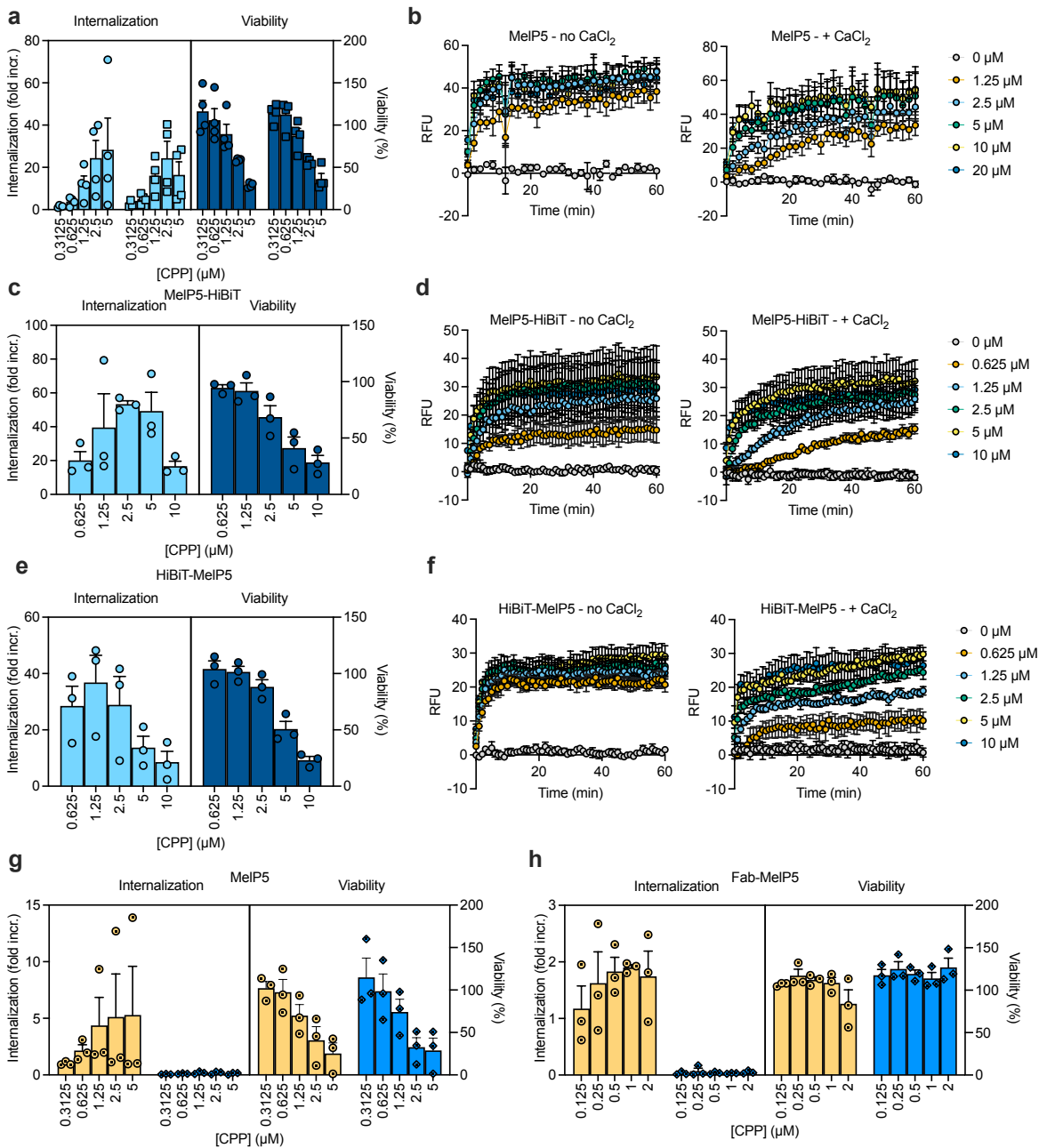

Supplement: S10 Fig — (a) Internalization of 100 nM (circles) or 1 μM (squares) HiBiT peptide, mediated by different concentrations of MelP5 in trans. Internalization is expressed as a fold increase over no MelP5 control wells, containing only HiBiT and a DMSO concentration equivalent to that in the MelP5 added wells. Viability is calculated relative to this control to control for any effect of DMSO. (b) Membrane damage mediated by MelP5, measured by an increase in PI fluorescence over time, with a background DMSO only control subtracted from each MelP5 concentration. (c, e) Internalization of HiBiT-MelP5 fusions. Results are expressed relative to a HiBiT only control. (d, f) Membrane damage mediated by MelP5 fusions to HiBiT, measured by PI fluorescence over time. Plots show DMSO background subtracted results. (g) Internalization of Fab fragments targeting gD (orange, circles) or transferrin receptor (blue, diamonds), mediated by different concentrations of MelP5 in trans. All Fabs have a C-terminal HiBiT peptide on the HC, for detection in the luciferase complementation assay. Results are expressed relative to a no MelP5, Fab only control. (h) Internalization of Fab fragments targeting gD (orange, circles) or transferrin receptor (blue, diamonds) with MelP5 fused to the C-terminus of the LC. All Fabs also contain a C-terminal HiBiT peptide on the HC. Results are expressed relative to a matching no MelP5 control Fab. For (a, c, e, g) bars show the mean, error bars show the SEM. For (b, d and f) symbols show the mean and error bars show the SEM. Replicates were as follows: (a) n = 4, (b-h) n = 3. A subset of data from these Supplementary Figures is reproduced in Figs 3–5 in the main manuscript to show optimal concentrations for each CPP and side-by-side comparisons of the different CPPs. (PDF) [file pone.0305848.s010.pdf]

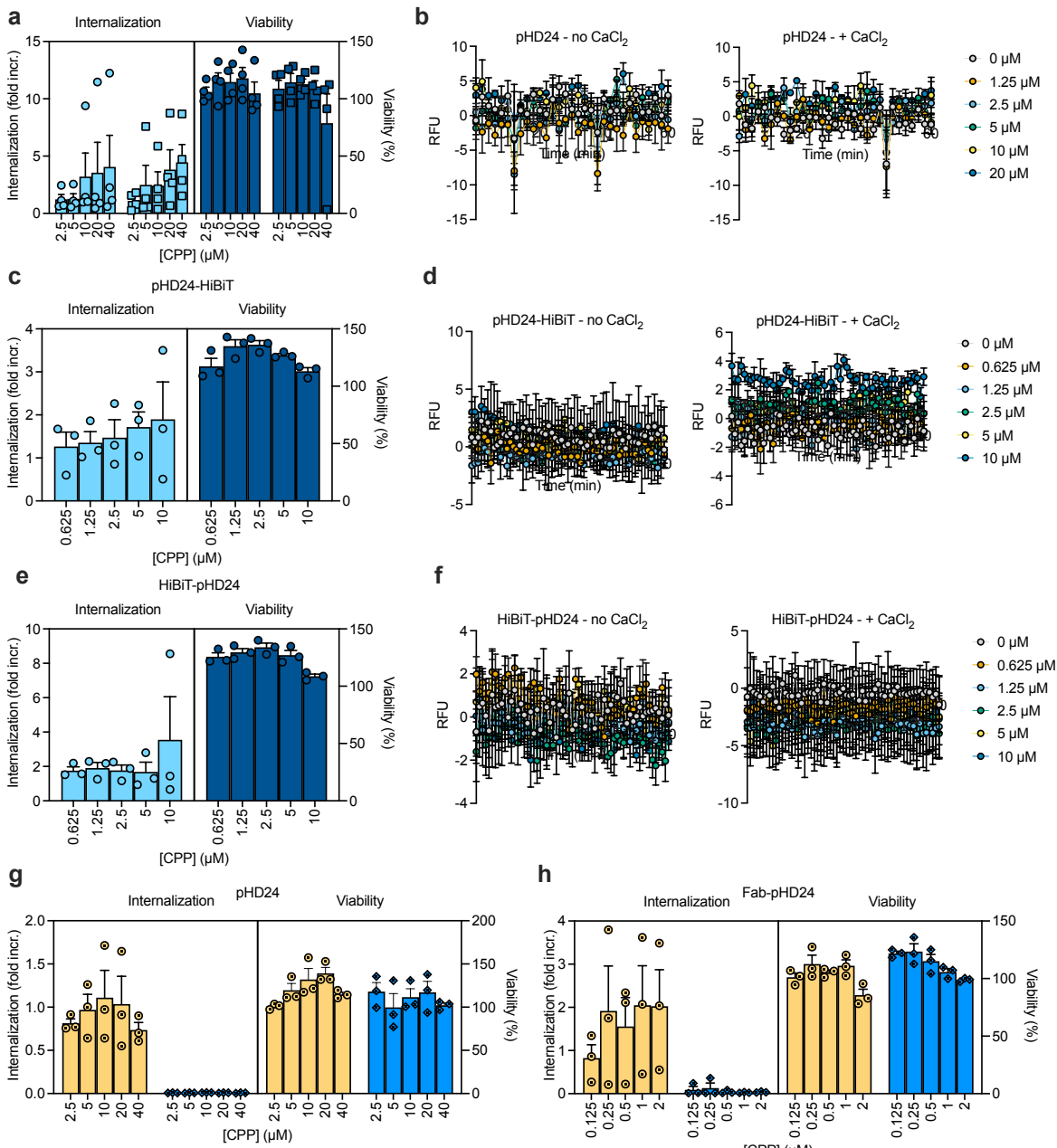

Supplement: S11 Fig — (a) Internalization of 100 nM (circles) or 1 μM (squares) HiBiT peptide, mediated by different concentrations of pHD24 in trans. Internalization is expressed as a fold increase over no pHD24 control wells, containing only HiBiT and a DMSO concentration equivalent to that in the pHD24 added wells. Viability is calculated relative to this control to control for any effect of DMSO. (b) Membrane damage mediated by pHD24, measured by an increase in PI fluorescence over time, with a background DMSO only control subtracted from each pHD24 concentration. (c, e) Internalization of HiBiT-pHD24 fusions. Results are expressed relative to a HiBiT only control. (d, f) Membrane damage mediated by pHD24 fusions to HiBiT, measured by PI fluorescence over time. Plots show DMSO background subtracted results. (g) Internalization of Fab fragments targeting gD (orange, circles) or transferrin receptor (blue, diamonds), mediated by different concentrations of pHD24 in trans. All Fabs have a C-terminal HiBiT peptide on the HC, for detection in the luciferase complementation assay. Results are expressed relative to a no pHD24, Fab only control. (h) Internalization of Fab fragments targeting gD (orange, circles) or transferrin receptor (blue, diamonds) with pHD24 fused to the C-terminus of the LC. All Fabs also contain a C-terminal HiBiT peptide on the HC. Results are expressed relative to a matching no pHD24 control Fab. For (a, c, e, g) bars show the mean, error bars show the SEM. For (b, d and f) symbols show the mean and error bars show the SEM. Replicates were as follows: (a) n = 4, (b-h) n = 3. A subset of data from these Supplementary Figures is reproduced in Figs 3–5 in the main manuscript to show optimal concentrations for each CPP and side-by-side comparisons of the different CPPs. (PDF) [file pone.0305848.s011.pdf]

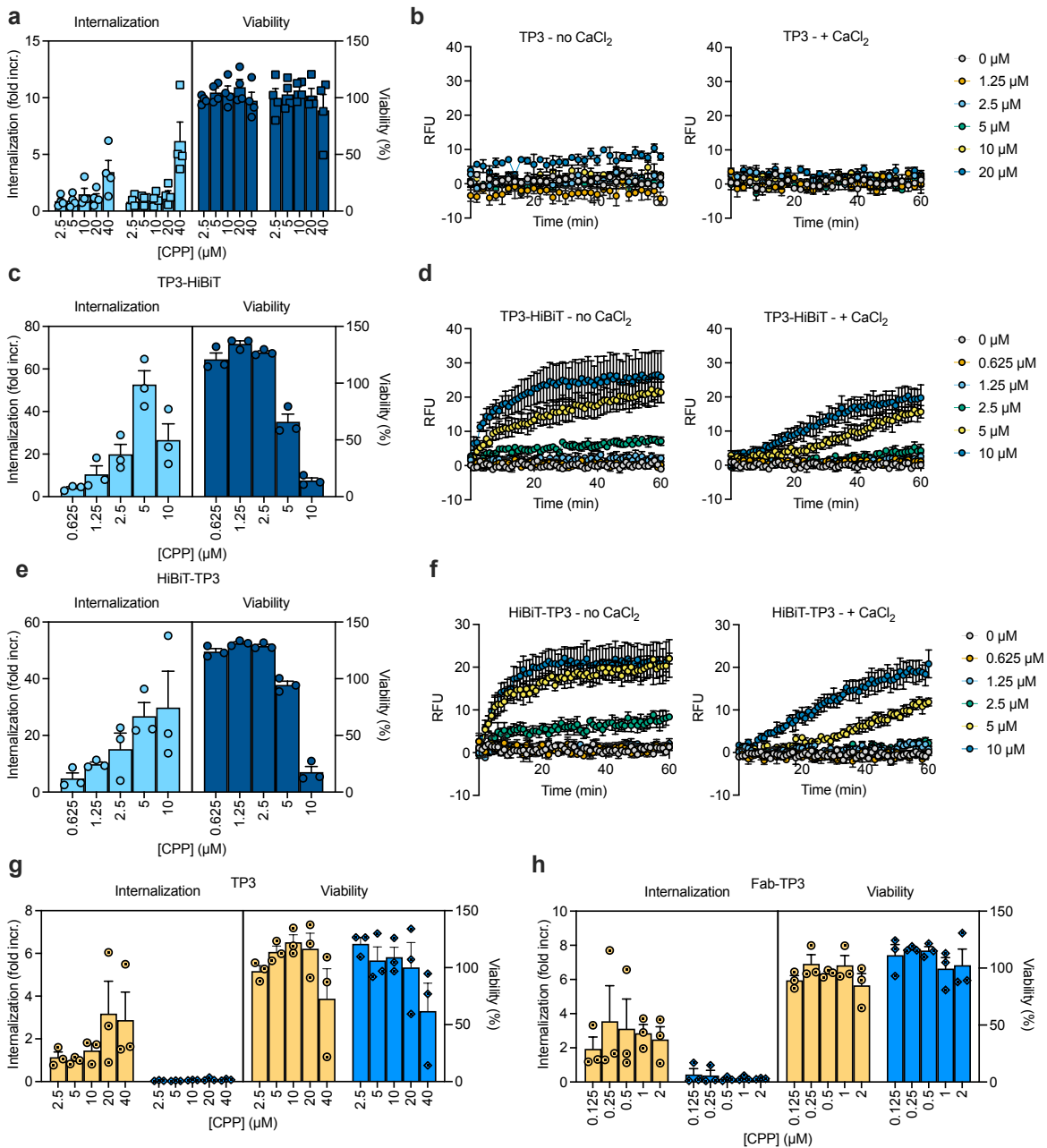

Supplement: S12 Fig — (a) Internalization of 100 nM (circles) or 1 μM (squares) HiBiT peptide, mediated by different concentrations of TP3 in trans. Internalization is expressed as a fold increase over no TP3 control wells, containing only HiBiT and a DMSO concentration equivalent to that in the TP3 added wells. Viability is calculated relative to this control to control for any effect of DMSO. (b) Membrane damage mediated by TP3, measured by an increase in PI fluorescence over time, with a background DMSO only control subtracted from each TP3 concentration. (c, e) Internalization of HiBiT-TP3 fusions. Results are expressed relative to a HiBiT only control. (d, f) Membrane damage mediated by TP3 fusions to HiBiT, measured by PI fluorescence over time. Plots show DMSO background subtracted results. (g) Internalization of Fab fragments targeting gD (orange, circles) or transferrin receptor (blue, diamonds), mediated by different concentrations of TP3 in trans. All Fabs have a C-terminal HiBiT peptide on the HC, for detection in the luciferase complementation assay. Results are expressed relative to a no TP3, Fab only control. (h) Internalization of Fab fragments targeting gD (orange, circles) or transferrin receptor (blue, diamonds) with TP3 fused to the C-terminus of the LC. All Fabs also contain a C-terminal HiBiT peptide on the HC. Results are expressed relative to a matching no TP3 control Fab. For (a, c, e, g) bars show the mean, error bars show the SEM. For (b, d and f) symbols show the mean and error bars show the SEM. Replicates were as follows: (a, b) n = 4, (c-h) n = 3. A subset of data from these Supplementary Figures is reproduced in Figs 3–5 in the main manuscript to show optimal concentrations for each CPP and side-by-side comparisons of the different CPPs. (PDF) [file pone.0305848.s012.pdf]

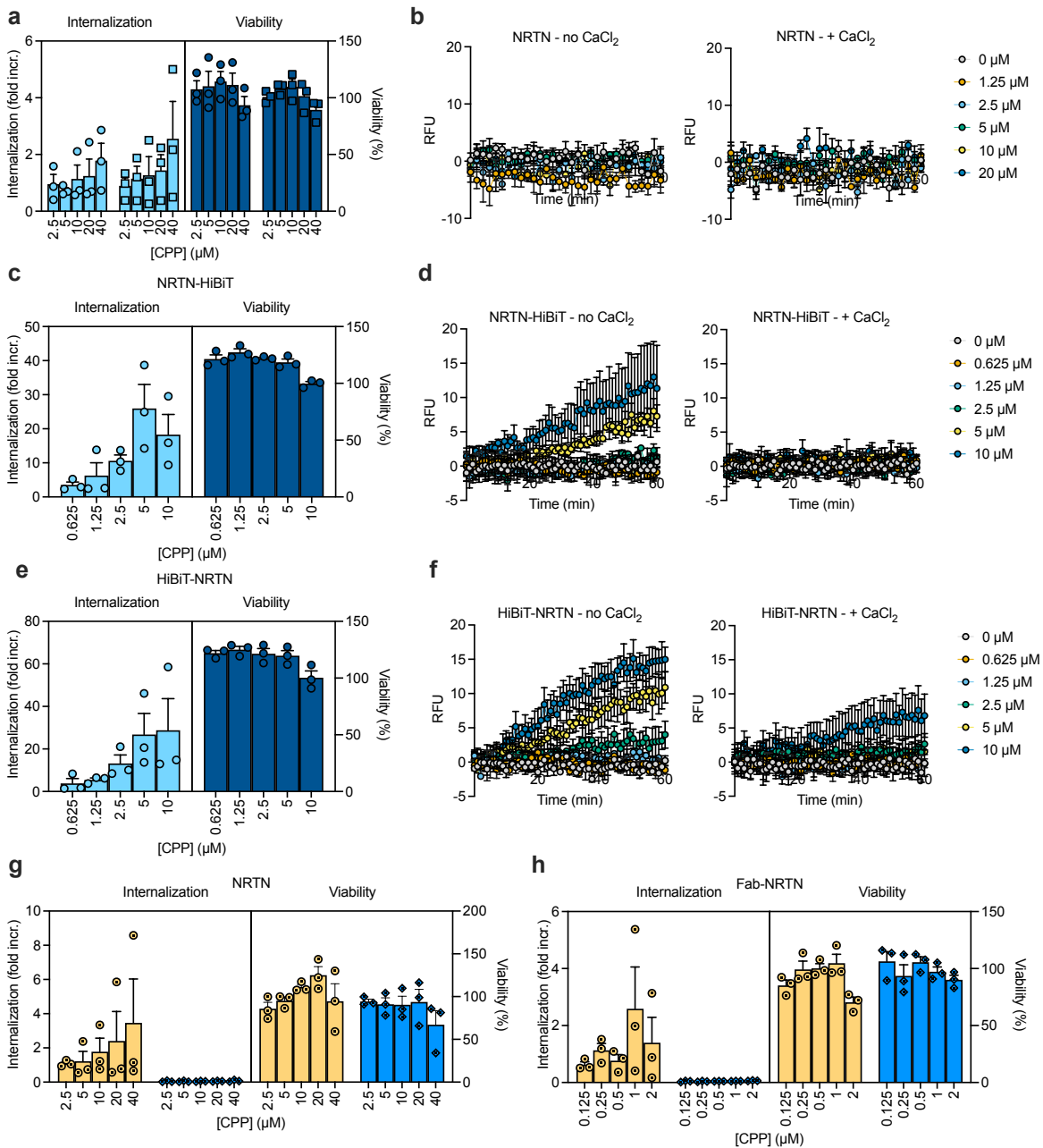

Supplement: S13 Fig — (a) Internalization of 100 nM (circles) or 1 μM (squares) HiBiT peptide, mediated by different concentrations of NRTN in trans. Internalization is expressed as a fold increase over no NRTN control wells, containing only HiBiT and a DMSO concentration equivalent to that in the NRTN added wells. Viability is calculated relative to this control to control for any effect of DMSO. (b) Membrane damage mediated by NRTN, measured by an increase in PI fluorescence over time, with a background DMSO only control subtracted from each NRTN concentration. (c, e) Internalization of HiBiT-NRTN fusions. Results are expressed relative to a HiBiT only control. (d, f) Membrane damage mediated by NRTN fusions to HiBiT, measured by PI fluorescence over time. Plots show DMSO background subtracted results. (g) Internalization of Fab fragments targeting gD (orange, circles) or transferrin receptor (blue, diamonds), mediated by different concentrations of NRTN in trans. All Fabs have a C-terminal HiBiT peptide on the HC, for detection in the luciferase complementation assay. Results are expressed relative to a no NRTN, Fab only control. (h) Internalization of Fab fragments targeting gD (orange, circles) or transferrin receptor (blue, diamonds) with NRTN fused to the C-terminus of the LC. All Fabs also contain a C-terminal HiBiT peptide on the HC. Results are expressed relative to a matching no NRTN control Fab. For (a, c, e, g) bars show the mean, error bars show the SEM. For (b, d and f) symbols show the mean and error bars show the SEM. Replicates were as follows: (a) n = 4, (b-h) n = 3. A subset of data from these Supplementary Figures is reproduced in Figs 3–5 in the main manuscript to show optimal concentrations for each CPP and side-by-side comparisons of the different CPPs. (PDF) [file pone.0305848.s013.pdf]

anti-gD

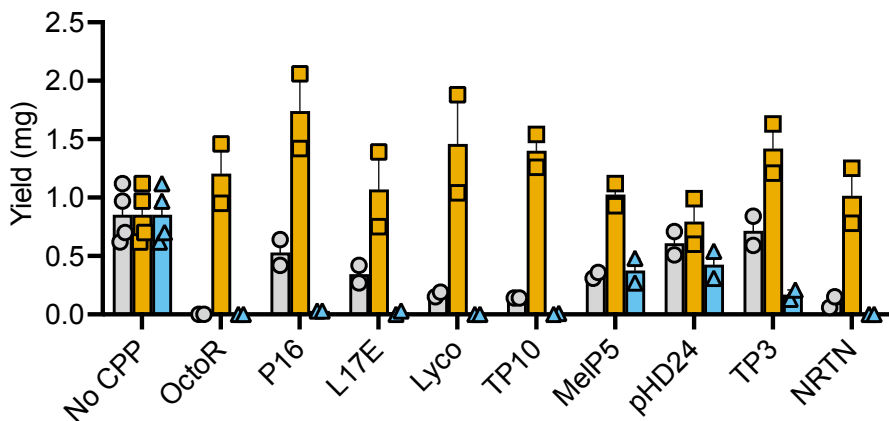

anti-TfnR<sup>1</sup>

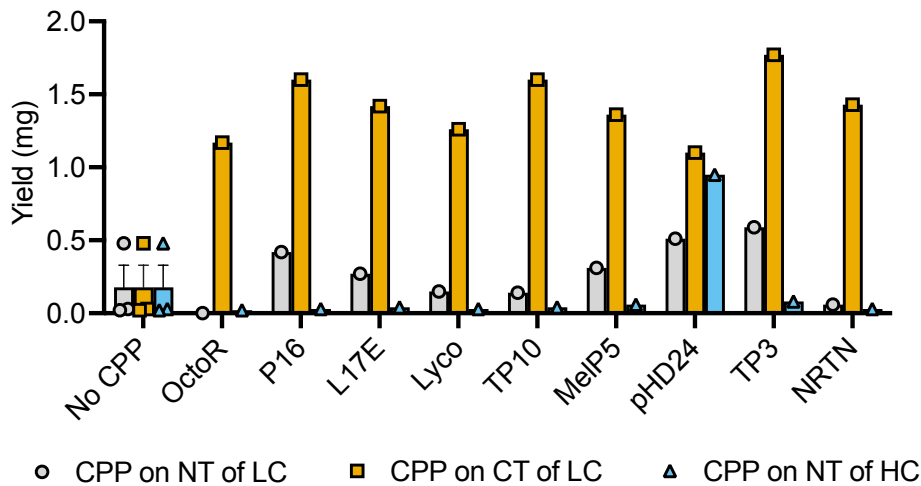

Supplement: S14 Fig — All yields are from 30 ml expressions, purified using a single-step affinity purification using CH1-XL resin. Individual replicates are shown. Bars show either a single replicate, or the mean of multiple replicates where available. For multiple replicates, error bars show the SEM. (PDF) [file pone.0305848.s014.pdf]
