# Supplementary material for: Plasma membrane damage limits cytoplasmic delivery by conventional cell penetrating peptides
Source: PLoS One. 2024 Sep 3;19(9):e0305848. doi: 10.1371/journal.pone.0305848 (PMC11371239; doi:10.1371/journal.pone.0305848)
Supplement: S16 Fig — Raw luminescence values for internalization are plotted on the left axis, raw luminescence values for viability (CTG) are plotted on the right axis. n = 2, bars show the mean. Fabs with the CPP on the LC N-terminus are shown in red, LC C-terminus in blue. (PDF) [file pone.0305848.s016.pdf]

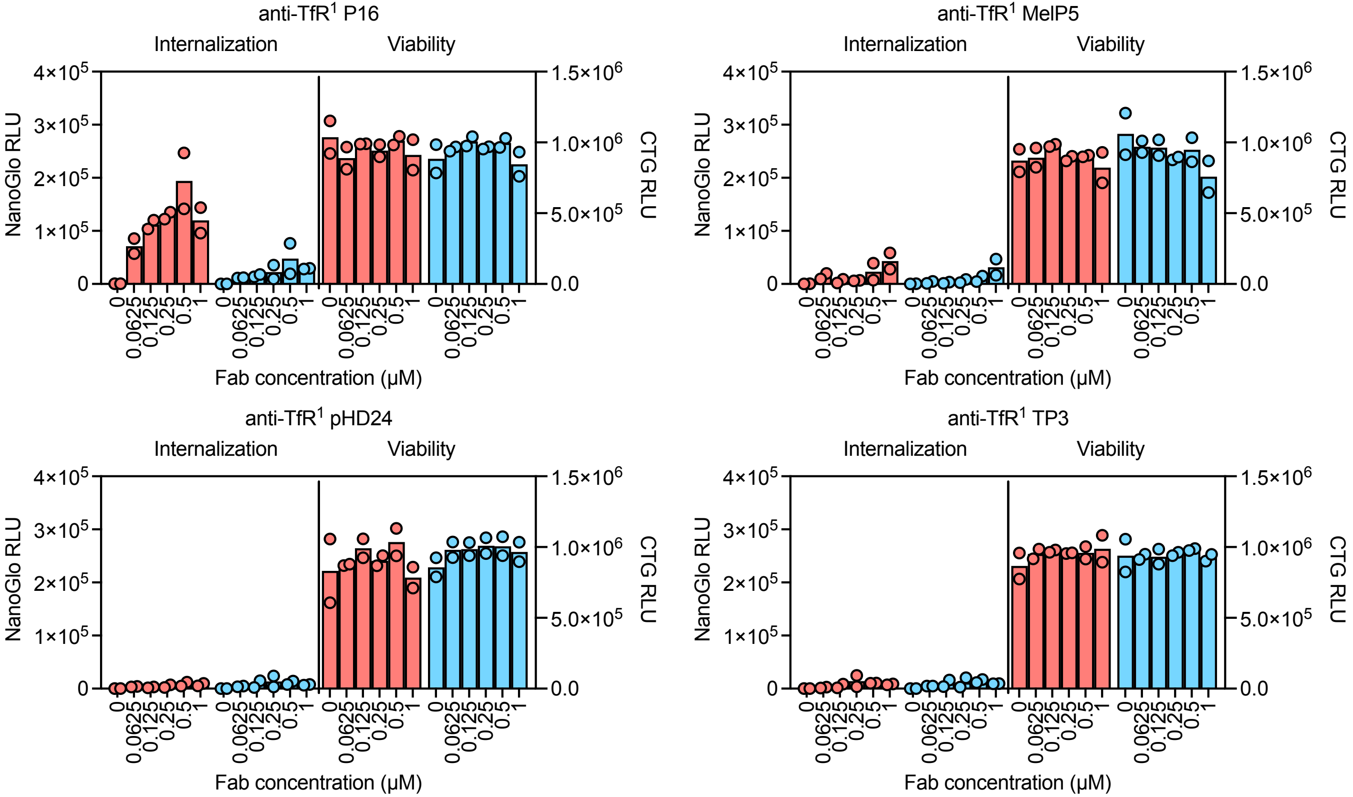
anti-TfR1 P16
Internalization
Viability
NanoGlo RLU
CTG RLU
4×10^5
3×10^5
2×10^5
1×10^5
0
1.5×10^6
1.0×10^6
5.0×10^5
0.0
Fab concentration (μM)
anti-TfR1 MelP5
Internalization
Viability
NanoGlo RLU
CTG RLU
Fab concentration (μM)
anti-TfR1 pHD24
Internalization
Viability
NanoGlo RLU
CTG RLU
Fab concentration (μM)
anti-TfR1 TP3
Internalization
Viability
NanoGlo RLU
CTG RLU
Fab concentration (μM)
