# Supplementary material for: Plasma membrane damage limits cytoplasmic delivery by conventional cell penetrating peptides
Source: PLoS One. 2024 Sep 3;19(9):e0305848. doi: 10.1371/journal.pone.0305848 (PMC11371239; doi:10.1371/journal.pone.0305848)
Supplement: S1 Table — (PDF) [file pone.0305848.s017.pdf]

S1 Table. Size comparison of broadly utilized cargo for assessing intracellular delivery of CPPs.

Cargo	Approximate Mass (Da) as surrogate for cargo size	Cargo has therapeutic potential
Fluorescent dye	500-1,500	no
siRNA	13,000	yes
Single-domain antibody (VHH) and antibody mimetics (e.g. DARPin)	15,000	yes
Green fluorescent protein	27,000	no
Fab	50,000	yes
IgG	150,000	yes
