# Supplementary material for: Plasma membrane damage limits cytoplasmic delivery by conventional cell penetrating peptides
Source: PLoS One. 2024 Sep 3;19(9):e0305848. doi: 10.1371/journal.pone.0305848 (PMC11371239; doi:10.1371/journal.pone.0305848)
Supplement: S1 File — (PDF) [file pone.0305848.s019.pdf]

Reporter protein sequences

```
>LgBiT_11S
MKHQHQHQHQHQHQMHQGSVFTLEDFVGDWEQTAAYNLDQVLEQGGVSSLLQNLAVSVTPIQRIVRSGE
NALKIDIHVIIPYEGLSADQMAQIEEVFKVVYPVDDHHFKVILPYGTLVIDGVTPNMLNYFGRPYEGIA
VFDGKKITVTGTLWNGNKIIDERLITPDGSMLFRVTINS
```

HQ tag
spacer
LgBiT_11S
